# The scoring of poses in protein-protein docking: current capabilities and future directions

**DOI:** 10.1186/1471-2105-14-286

**Published:** 2013-10-01

**Authors:** Iain H Moal, Mieczyslaw Torchala, Paul A Bates, Juan Fernández-Recio

**Affiliations:** 1 Joint BSC-IRB Research Program in Computational Biology, Life Science Department, Barcelona Super computing Center, Barcelona 08034, Spain; 2 Biomolecular Modelling Laboratory, Cancer Research UK London Research Institute, London WC2A 3LY, UK

**Keywords:** Docking, Scoring functions, Binding energy, Ranking, SwarmDock

## Abstract

**Background:**

Protein-protein docking, which aims to predict the structure of a protein-protein complex from its unbound components, remains an unresolved challenge in structural bioinformatics. An important step is the ranking of docked poses using a scoring function, for which many methods have been developed. There is a need to explore the differences and commonalities of these methods with each other, as well as with functions developed in the fields of molecular dynamics and homology modelling.

**Results:**

We present an evaluation of 115 scoring functions on an unbound docking decoy benchmark covering 118 complexes for which a near-native solution can be found, yielding top 10 success rates of up to 58%. Hierarchical clustering is performed, so as to group together functions which identify near-natives in similar subsets of complexes. Three set theoretic approaches are used to identify pairs of scoring functions capable of correctly scoring different complexes. This shows that functions in different clusters capture different aspects of binding and are likely to work together synergistically.

**Conclusions:**

All functions designed specifically for docking perform well, indicating that functions are transferable between sampling methods. We also identify promising methods from the field of homology modelling. Further, differential success rates by docking difficulty and solution quality suggest a need for flexibility-dependent scoring. Investigating pairs of scoring functions, the set theoretic measures identify known scoring strategies as well as a number of novel approaches, indicating promising augmentations of traditional scoring methods. Such augmentation and parameter combination strategies are discussed in the context of the learning-to-rank paradigm.

## Background

Protein-protein interactions are elemental to almost all biological processes. The atomic-resolution annotation of protein interaction networks can give insights into the kinetics [1-5], thermodynamics [6-10] and organisation [11-13] of the complex systems they constitute, as well as human disease [14,15]. The 3D structures of a protein-protein complex can be used to estimate the effect of mutations [16-21], and thus for protein design [22-29] and determining the functional consequences of mutations associated with diseases (for instance [30]). Further, protein-protein interactions are receiving considerable attention as targets for rational drug design [31-33] and as therapeutic agents [34-37], both endeavours in which structural information is invaluable. However, in spite of this importance, the rate at which the structures of protein complexes are being solved experimentally lags far behind the rate at which interactions are being discovered. As such, there is a pressing need to fill this ever-growing gap with models derived through computational means such as docking [38-45] and post-docking analysis [46,47].

Despite over three decades of investigations, protein-protein docking remains an unsolved problem. Out of the two critical challenges in docking, the first is sampling, especially in cases with large conformational flexibility. The second, scoring, is the topic of this paper, and is concerned with identifying the correct docking conformations. Scoring attempts to identify the lowest energy poses, and is thus related to the problem of predicting benchmarks of experimental ΔG and ΔΔG values [48,49], an area in which further work is required [50,51]. This is usually achieved by ranking the structures that are generated by docking algorithms, and a number of different approaches have been applied to this problem. These range from composite scoring functions using a linear combination of terms, usually models of the underlying physical phenomena at play [52-65], to methods derived from the statistics of structural databases [63,66-70], docking decoys [71-75], experimental binding energies [76], methods based on interface composition and geometry [77-80] or complementarity [81-90], methods based on machine learning [91-97], and methods which account for the characteristics of the binding funnel [98-102]. These approaches span a range of resolution from residue-level to atomic. Further, potentials derived from the field of homology modelling can show promise when applied to interactions [103-105], yet many such methods have not yet had their ability to rank docked structures ascertained.

A large-scale evaluation of the ability of 115 different metrics to rank docked poses using a set of docking decoys generated from the protein-protein docking Benchmark 4.0 [106], using the SwarmDock algorithm [107-109], is presented here. These metrics include docking scores, their constituent terms, molecular mechanics energy functions and methods developed by the protein folding community. We also analyse the union, symmetric difference and relative complement between sets of complexes identified by different methods so as to give an indication of the amount of mutual information embedded in pairs of scoring functions, and the potential for different methods to be combined together synergistically. The results of these investigations give an indication of which approaches are most successful and suggest a number of promising future directions for the improvement of scoring functions.

## Results and discussion

### Both docking and folding potentials can rank docked poses

**Figure 1:**
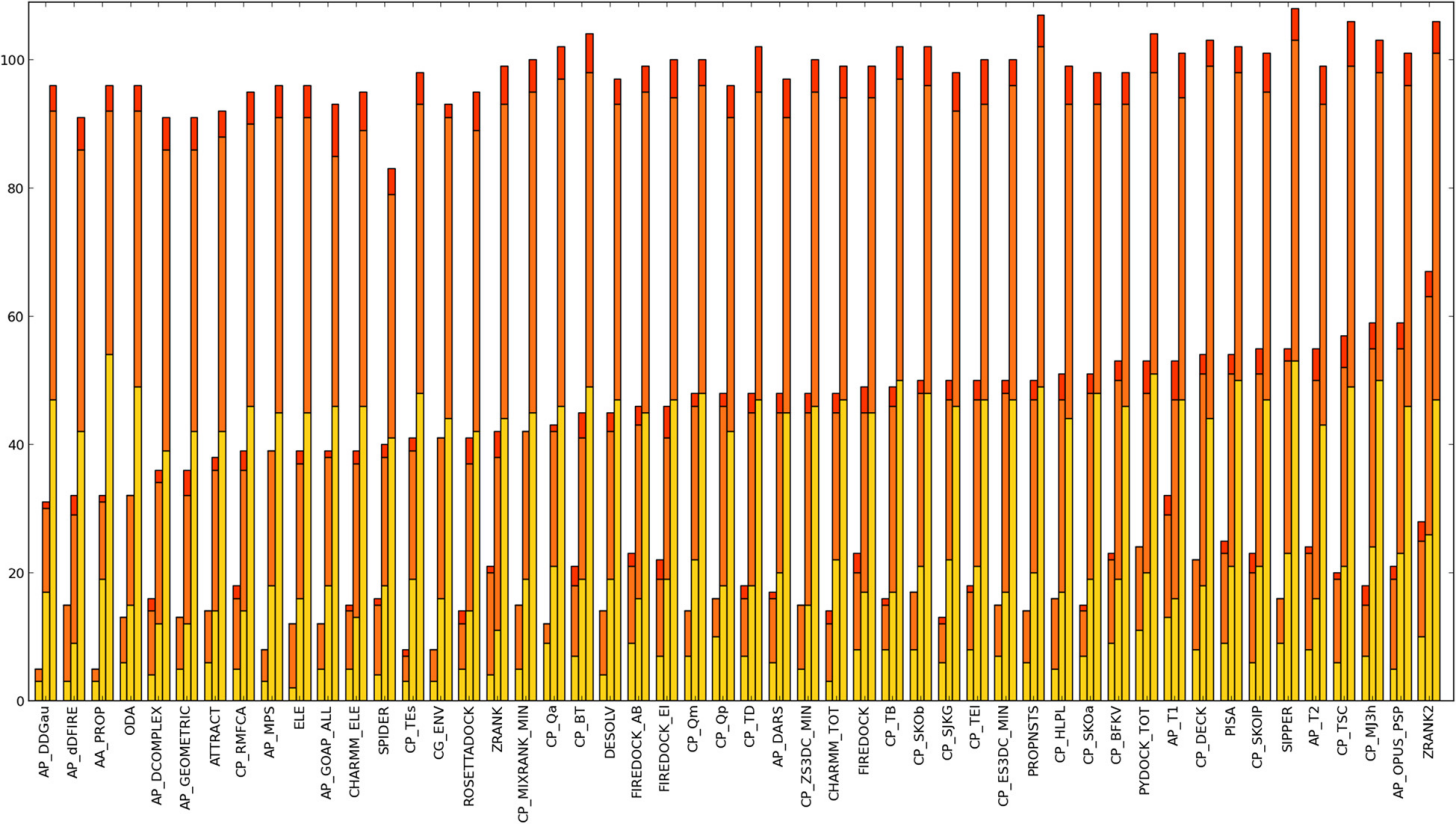
**The success rates for the highest performing scoring functions.** The success rates for the highest performing scoring functions. The number of complexes for which an acceptable or better solution could be found in the top 1, top 10 and top 100 solutions was calculated for each scoring function, and the best 40 scoring functions for each measure were selected. Acceptable quality solutions are shown in yellow, medium quality solutions in orange, and high quality solutions in red for the three measures (top 1 left, top 10 middle, top 100 right). The functions are ordered by top 10 success rate.

The results for the highest performing scoring functions are shown in Figure 1, ordered by top 10 acceptable or better success rate. Numerical values for all the scoring functions are shown in Additional file 1: Table S1, and results ordered by top 1 and top 100 success rates shown in Additional file 1: Figures S1 and S2. Regardless of rank, a medium or better solution could be found for 53% of the complexes, and a high quality for 7%. When ranked and clustered, an acceptable or better solution could be found in the top 100 for up to 92% of the complexes. For top 10 ranked solutions, overall success rates of up to 58% were observed, which dropped to 27% when only the top ranked solution was considered. As expected, methods specifically designed for protein-protein docking feature prominently, with several docking potentials [74,75], pyDock [54], SIPPER [67], DECK [72], PISA [110] and in particular ZRANK2 [58], showing a very good ability to discriminate near natives from incorrect decoys. The SKOIP intermolecular contact potential [111], which has not been optimised for docking, also performed very well. One consideration that should be made when interpreting the results for the docking-specific methods is that, while these models have been trained on docked structures, none have been specifically trained using SwarmDock decoys. Specifically, using a scoring function outside of the domain used to train it may result in false positives (*e.g.* when encountering an interface with more highly optimised hydrophobic contacts compared to the examples used to train the score) and false negatives (*e.g.* disallowing a near-native structure due to clashes, when the search method for which the scoring function was designed would not produce such contacts). Although SwarmDock does not permit clashes in its solutions, these examples should serve to demonstrate that performance may differ markedly when a different docking algorithm is used, and may be higher on structures generated using the same methods as for training. Thus, the evaluation here is not the same as evaluating the whole docking protocol in the context of the search function used to generate the structures. Nonetheless, the fact that all the docking-specific methods evaluated work well at ranking the SwarmDock decoys indicates that methods designed specifically for one algorithm can also be used to rank poses generated by a different algorithm. This should come as no surprise, as all scoring functions ultimately attempt to identify the structure with the lowest binding energy, and the energy of a given configuration depend only on its coordinates, not on the method used to generate those coordinates. Often, the optimisation acts only to balance the energetic terms. Thus there is a certain degree of interchangeability of scoring functions, and the results can provide information on how well these scoring functions identify the structural aspects that confer affinity, as long as the above caveat is taken into account.

**Figure 2:**
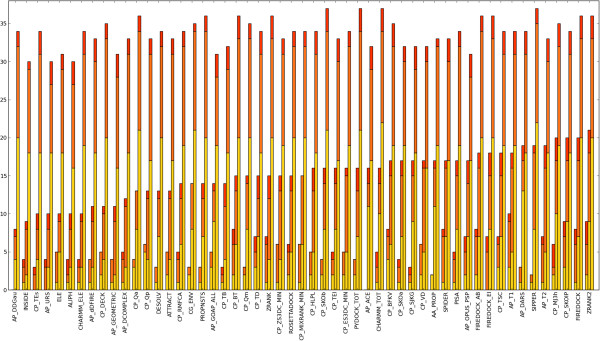
**The success rates for the highest performing scoring functions using the Benchmark 4.0 update.** The success rates for the highest performing scoring functions using the Benchmark 4.0 update. These are the new complexes which were not present in previous versions of the benchmark. The performances are displayed and ordered as in Figure 1.

Another consideration is that the performance may be overestimated due to the methods being trained on complexes in the test set. To mitigate such biases and make fair comparisons, the scoring functions were evaluated again using only the updated structures in the Benchmark 4.0, a set which was not used in the training of any of the scoring functions. The results of this are shown in Figure 2. None of the highest performing docking-specific methods perform poorly, indicating that none are drastically over-fitted, while FireDock [59], DARS [73] and SPIDER [78] join the methods above as also being of particular merit. Interestingly, many of the top methods are coarse-grain.

The results harbour a number of surprises. For instance, the fine-grain weighted RosettaDock scoring function performs comparably to the coarse-grain Rosetta environment potential, CG_ENV [112], and the MixRank strategy does not perform as well as its constituent S3DC potential [69]. However, perhaps the greatest surprise is the capability for some of the folding potentials to identify near-native solutions. Two methods in particular stand out. The first is the OPUS_PSP potential, a side-chain only orientation-dependent statistical contact potential in which residues are decomposed into rigid and planar chemical moieties [113]. It would be intriguing to see the performance of this potential after training it as an intermolecular potential with crystal structures or near-native docked solution as observations, and/or with docking decoys as the reference state, should a sufficient number of complexes become available. The second is MJ3h, a coarse-grain statistical contact potential which has been corrected for water-to-protein transfer energy [114]. This very simple potential outperforms many more complex scoring functions and, remarkably, has a good ability to distinguish the high quality solutions from other near-native poses.

### Difficulty and quality dependent efficacy suggests a need for case-dependent scoring functions

To ascertain whether different scoring methods have different performances depending on difficulty, the analysis was repeated using only the easier cases, the rigid-body category of the Benchmark 4.0, and the harder cases. As the medium and difficult categories contain fewer complexes, and near-native solutions were found less frequently, they are considered together. The results for the rigid-body cases are shown in Additional file 1: Figures S3, S4 and S5, with high-performing methods similar to those for the whole benchmark. As expected, docking of these cases was more successful than for the flexible cases, with top 1, top 10 and top 100 acceptable or better success rates of up 30%, 63% and 93% of cases where such a solution is available. For the flexible cases, with results shown in Additional file 1: Figures S6, S7 and S8, only up to 4 complexes were top ranked by any method, too few to reliably distinguish between the different scores. However, for the top 10 and top 100 solutions, success rates of up to 36% and 86% could be achieved. It can be seen that for these complexes, the highest performing methods are highly enriched with coarse-grained scoring functions. This is consistent with the difficulty in correctly predicting the specific atomic contacts for the most flexible cases. While the use of smoothed and coarse-grained functions for the compensation of conformational uncertainty is a common docking strategy [55,61,62,67,115], the differential scoring performances shown here suggest a role for flexibility-dependent scoring functions, that may be used in conjunction with methods for predicting flexibility [116-120].

In order to further investigate how the ability to score a docked pose depends on the quality of the pose, we investigated the conditional probability of finding a structure of at least a given quality or better given that a solution of at least that quality exists. While an insufficient number of high quality solutions were generated to derive meaningful statistics, this was undertaken for the medium and acceptable quality for all 122 methods, with the results given in Additional file 1: Table S2. When defining a solution as found if it appears in the top 10, 66% of the methods had a greater conditional probability of finding a medium or better solution than an acceptable or better (76/115), indicating a slightly higher success rate for the higher quality solutions. However, the methods evaluated here are biased towards coarse-grain models. When evaluating only the models prefixed with ‘AP_’ , all of which are at or near atomic resolution, this figure rises to 71% (15/21), demonstrating that the high-resolution scoring functions preferentially identify the higher quality solutions. For the scoring functions prefixed with ‘CP_’ , all of which are at residue resolution, the figure is 64% (34/53), indicating a lesser preferential ability to identify the higher quality solutions. These results further suggest that different scoring strategies are best employed for different docking difficulties.

### Differential performance identifies existing and novel scoring strategies

**Figure 3:**
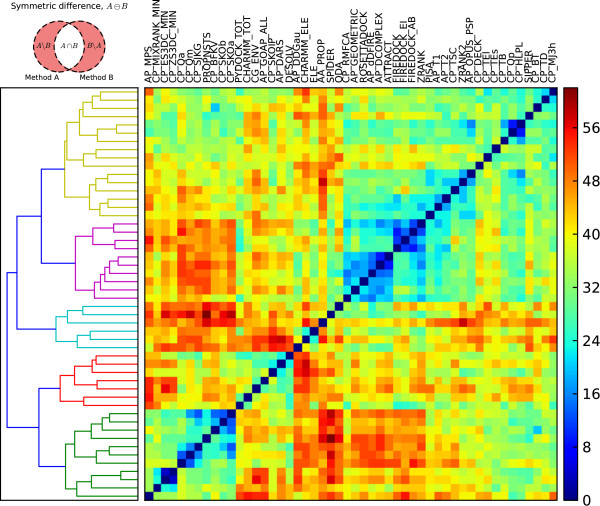
**The cardinalities of symmetric differences for pairs of high performing scoring functions.** The cardinalities of symmetric differences for pairs of high performing scoring functions. Matrix indices were determined by complete-linkage clustering of the scoring functions, with dissimilarity defined by the cardinality of the symmetric difference sets. The corresponding dendrogram is shown on the left, with the cophenetic distance given by the U-link height. High cardinalities indicate greater ability for the scoring function pairs to identify near-native poses of acceptable or better quality in the top 10 models for different subsets of complexes.

We wished to determine whether the subset of complexes found by any of the methods was significantly different from the subsets found by the other methods. To investigate whether different methods are capable of correctly identifying near-natives in different subsets of the complexes, we looked at all pairs of the methods given in Figure 1. For each pair, we calculated the union (*i.e.* set of complexes found by either methods), symmetric difference (*i.e.* complexes found by only one of the methods) and relative complement (*i.e.* complex found by one method but not the other) of the sets of complexes which were ranked as acceptable or better in the top 10. The numerical results for this analysis are given in Additional file 1: Table S3. While a combined scoring function would not necessarily be able to identify all the correctly docked pose identified by either of the individual scoring functions, nor necessarily miss structures missed by both methods, the cardinalities of the resultant sets can give insights into the extents to which deficiencies in one scoring function may be compensated by another. For the union and symmetric differences, the larger the size of the resultant sets, the greater the ability of the two methods to identify different complexes. The symmetric difference data is shown in Figure 3. The pairs of scoring functions with highest cardinality are those containing the least mutual information, and are thus most likely to work synergistically together. They suggest some intriguing strategies, including some that have already been developed and applied, as well as novel approaches that might merit from further investigations. For instance, of the 70 complexes which are found using either ODA (33) or PROPNSTS (51), 56 of them are found by only one method and not the other (the other 14 are found by both of them). This indicates that they are detecting different aspects and would work well together. We know this to be true, as the ODA score represents residue and geometry specific exposure of hydrophobic surface [121,122], and PROPNSTS represents chemical complementarity of amino acid pairs [67]. Indeed, the combination of these two terms is the basis for the SIPPER scoring function [67], which routinely performs better than either of the two methods on their own, and can identify near native solutions of acceptable or better quality for 56 complexes. Another common pair of score types with high cardinality is the mix of electrostatics and statistical potentials. Again, this combination is already exploited in the high performing methods ZRANK [57], ZRANK2 [58] and FireDock [59]. One type of term that is not currently included in the ZRANK and FireDock methods, however, are coarse-grain pair potentials. These results suggest that coarse-grain potentials are capable of finding different subsets of complexes and thus could further enhance these methods. The most promising pairs of methods suggested by the symmetric difference measure, however, are mixtures of SPIDER [78] and other approaches. SPIDER is a novel coarse-grain procedure in which the interfaces of known complexes were decomposed into networks and common motifs found by subgraph mining. When used for scoring, the docking decoys are similarly decomposed and ranked according to the presence of network motifs. While SPIDER is not exceptional on its own, it is good at finding structures which are missed by the other methods. This suggests that this method could be powerful when combined with other techniques. Interestingly, SPIDER distinguishes itself from the other methods in that it explicitly considers multi-body interactions, as opposed to only pairwise interactions, suggesting that other multi-body methods could yield equally promising avenues of exploration. Another interesting aspect of the symmetric difference data is how the scoring functions cluster by similarity. Consider the second principal clusters, shown as the purple subtree in the dendrogram of Figure 3. This cluster splits into two subclusters. The corresponding blue squares indicate that RMFCA, GEOMETRIC, RosettaDock, dDFIRE, DComplex, and ATTRACT are very similar in terms of the near-natives they can correctly identify. In turn, they are also similar to FireDock and ZRANK, which are even more similar to one another. Looking at the first principle cluster, we find similarities between these functions and other methods including ZRANK2, SIPPER, PISA, DECK, other docking potentials and the two highest performing homology modelling potentials, OPUS_PSP and MJ3h. Moreover, these two clusters contain all but one of the composite scoring functions and most of the atomic resolution functions. This makes a stark contrast to the last principle cluster, shown as the green subtree in Figure 3. With only one exception, this is made up only of coarse-grain potentials. These interactions tend to have high cardinalities when paired with the second principle cluster. This is, in part, due to the generally higher performance of the methods in the second cluster, but also due to the fact that these potentials are capable of finding the higher flexibility cases and lower quality near-native solutions, as noted in the previous section. The third and fourth principal clusters, shown in cyan and red, contain an assortment of potentials, including the CHARMM energy, two electrostatics models (ELE and CHARMM_ELE), two residue-level desolvation terms (ODA and CG_ENV), SPIDER, the DARS and GOAP potentials, and a potential extracted from energy changes upon mutation (AP_DDGau). These disparate potentials have high cardinalities amongst themselves and with the functions in the other principal clusters, indicating that they may be capturing aspects which are overlooked by the other functions.

**Figure 4:**
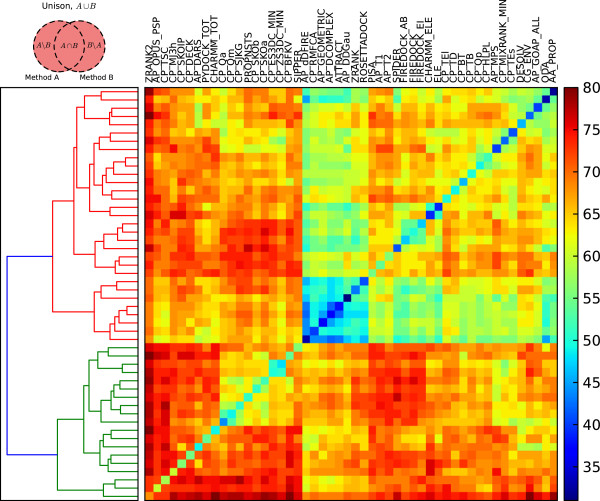
**The cardinalities of unions for pairs of high performing scoring functions.** The cardinalities of unions for pairs of high performing scoring functions. Clustering was performed as described in Figure 3, with the union defining the matrix. High cardinalities indicate that if a scoring function could be created from the two methods capable of identifying all the near natives correctly identified in the top 10 models for both methods, then it would identify a large proportion of the benchmark complexes.

While the cardinalities of symmetric difference are highly informative, especially when the methods being compared have comparable success rates, focussing on the differences between methods only gives some of the picture. For instance, large symmetric difference sets can be observed when comparing a very high-performing scoring function with a less successful method, not due to their synergistic value but because of deficiencies in the latter. Further, two pairs of methods could have equally sized symmetric differences yet have significantly different success rates due to differences in their intersection. However, such overlap is desirable as it indicates that the two methods can reinforce one another. For these reasons, we investigated the cardinalities of two different sets, the union set and the relative complement. The union data is shown in Figure 4. The data forms two principal clusters shown as red and green subtrees in the dendrogram of Figure 4. The first consists of methods which identify near natives in similar sets of complexes, and thus combining their sets amongst themselves does not largely expand the range of complexes correctly identified. The second cluster consists of the very high performing scoring functions, in particular the two subclusters which form the first nine scoring functions in the bottom left corner, which can be significantly enhanced if combined with each other and with many of the other functions. Within these two subclusters, one of the methods that performs the least on its own is the total CHARMM energy, yet this it is this method which makes the greatest unions within this cluster, particularly with ZRANK2, AP_OPUS_PSP and CP_TSC, suggesting that this energy function can complement these highly performing methods well. Other intriguing pairs within this cluster include CP_TSC with CP_SKOIP, both very computationally efficient contact potentials, ZRANK2 with CP_MJ3h, and AP_DARS with CP_TSC and ZRANK2. The first primary cluster, corresponding to the red subtree, consists of all the other methods, with great variation amongst themselves and with the second clusters. Within this cluster, the potentials which tend to form the highest cardinalities are PISA, AP_T1, AP_T2, SPIDER, the FireDock potentials and the CHARMM electrostatics potential, all but one of which are atomic resolution. These potentials also form high cardinalities when combined with the potentials in the second principle cluster, particularly with the coarse-grain potentials running from CP_Qa to SIPPER. Also of note are the Rosetta coarse-grain environment potential, CG_ENV, the DESOLVE solvation term and the AP_MPS potential.

**Figure 5:**
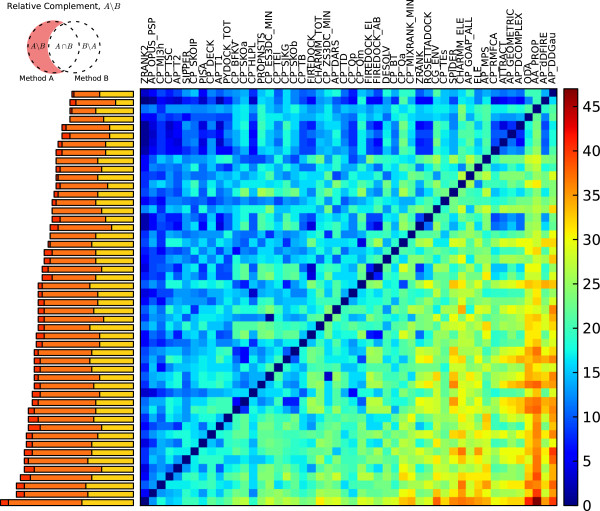
**The cardinalities of relative complements for pairs of high performing scoring functions.** The cardinalities of relative complements for pairs of high performing scoring functions. Indices are ordered by individual top10 acceptable or better success rate, as shown in the leftmost histogram, with acceptable, medium and high quality success rates shown in yellow, orange and red respectively. This matrix indicates the extent to which the method corresponding to each column can benefit from being able to identify the near-native solutions identified by the methods corresponding to each row. Equivalently, each row indicates the extent to which its method could contribute to the methods in each respective column.

The third and final set theoretic comparison method used is the relative complement, as shown in Figure 5. This asymmetric method can be seen as a decomposition of the symmetric difference measure. As the results are ordered by individual success rates, it can be clearly seen that the highest performing methods have the least to gain should they be able to identify the near-natives identified by the other methods (blue left hand side), and *vice versa* (red right hand side). This visualisation allows the identification of the methods which could contribute the most to other methods, in general, by finding rows with incongruously high values. These rows include some methods that have already been identified, such as ELE, CHARMM_ELE, CHARMM_TOT, SPIDER, AP_DARS, FIREDOCK and AP_MPS. Further, it can identify methods which could be used to further improve the already high performing scoring functions, by finding incongruously high values in the leftmost columns. For instance, it also suggests that the ZRANK2 method could be combined with CHARMM_TOT or CG_ENV, or that CP_MJ3h could be combined with ZRANK2, CP_TSC or CHARMM_ELE, or AP_OPUS_PSP with CP_BFKV. More significantly, CP_TSC could be profitably combined with a number of methods, such as CP_DECK, PYDOCK_TOT, CP_SKOa, CP_BVKV, SIPPER, CP_SKOIP, CP_SJKG, CHARMM_TOT, AP_DARS, CG_ENV, CP_Qm or CP_Qa.

## Conclusions

Here we have evaluated 115 different scoring functions using a recent docking benchmark [106], yielding acceptable or better solutions in the top 10 for up to 58% of cases. All of the docking-specific scoring functions evaluated performed well, vindicating a range of approaches, including pair potentials trained with docking decoys [72-75,110], composite scoring functions [54,57-59,61,67,112], and a novel approach based on the identification of common motifs in interacting residue networks [78]. Interestingly, some methods taken from the field of homology modelling also performed exceptionally well. In particular, two methods stood out. The first is a simple residue contact potential [114], which can be used to quickly evaluate thousands of structures [123], and thus would be suitable for the initial filtering of poses determined with algorithms that generated a large initial set of decoys, such as Fourier transform docking [89]. The second is a novel statistical potential [113], with accuracy that may be further enhanced if reparameterised as an intermolecular potential or using docking decoys.

We have only considered complexes for which SwarmDock could generate near-native poses. Nevertheless, for at least a third of the Benchmark 4.0, acceptable solutions were generated but not identified by any single scoring function on its own. However, near-native solutions missed by one method were often found by different methods. For instance some functions, particularly the coarse-grained, were better at identifying correct poses for the more difficult cases and where the quality of the docked solutions was lower. On the other hand, atomic potentials were superior for the less flexible cases and higher quality solution. This indicates that flexibility prediction could be used for the selection of the most appropriate scoring functions on a case-by-case basis, or incorporated into the scoring scheme. To investigate which potentials could be combined together, we identified pairs of scoring functions capable of correctly identifying near-natives in different subsets of the benchmark. To achieve this, we used three different set operators, which give complementary pictures of the data. This analysis identified a number of general strategies, such as the combination of amino acid propensities with hydrophobic burial, statistical potentials with electrostatics, and atomic-resolution functions with residue-level potentials. It also allowed the identification of individual methods which appear to capture aspects missed by traditional scoring functions, such as the SPIDER algorithm and the Rosetta environment potential. As SPIDER captures multi-body interactions, and these have not received significant attention in the field of protein-protein docking, this indicates computational characterisation of cooperative interactions across the interface should be a focus of future research. Finally, these methods also identify specific combinations of terms which may be promising, such as merging the CP_TSC potentials with the AP_DARS potential. For the scoring functions evaluated here, SwarmDock decoys were used. As the scoring functions are easily available, other groups could extend the analysis using structures generated with their own algorithms. However, the fact that methods optimised using different decoys sets still perform well on these structures indicates that scoring functions can be transferred from one docking algorithm to another. Subsequently, the insights garnered here should still apply.

Although this work suggests promising combinations of terms, we have not yet considered how these terms can be combined. A common approach is to take them in linear combination. This makes sense as a first approximation when the terms consist of energy models of physical phenomena, due to the additivity of thermodynamic cycles. However when physical phenomena are coupled, or when features are used that are not rooted in physical phenomena, non-linear relationships between the terms and their utility in ranking arise. Further, it would be desirable to account for heterogeneous data sources, such as predicted flexibility measures as suggested here, but also terms such as sequence conservation data, cluster sizes or agreement with experimental data, or polynominal features such as complex type or, if attempting to merge docking solutions from different algorithms, the provenance of each pose. In these cases, flexible machine learning algorithms capable of inferring these relationships can be used to tailor scoring functions to the structures produced by the sampling methods [124], and indeed examples of this approach can be found in the literature [91-97]. Surprisingly, however, all of these examples have treated the ranking of docked poses not as a ranking problem but as a classification problem, resulting in two issues. Firstly, they are narrow in terms of the models that they use. For instance, when ranking is undertaken in the pointwise approach, it can be reformulated as a classification, regression or ordinal classification problem [125-128]. Similarly a pairwise ranking approach can be formulated as pairwise classification or pairwise regression [129-136], for instance where a model is trained on restraints derived from the fact that acceptable docking poses are superior to incorrect poses, medium are superior to acceptable and incorrect poses, and so on. Further, listwise ranking methods could be employed where, instead of viewing each pose independently and assigning it a score, and instead of comparing pairs of poses to determine which is superior, the whole list is ranked simultaneously as an inseparable set [137-142]. The exploration of how docking ranking performs when reformulated along these lines remains to be seen. The second issue is that current machine learning based docking scoring methods do not directly address the needs of those who wish to rank docked solutions. For instance, they fail to distinguish high quality solutions from those that are merely acceptable. Additionally, for the purpose of docking, the difference between a top ranked pose and a pose ranked 20^th^ is much greater than the difference between poses ranked 101^st^ and 120^th^, and this should be reflected in the associated loss function. We suggest that terms such as those explored in this work should be combined in a way which more closely resembles how search engines rank documents. Just as page ranking strives to order lists of documents according to relevance with the most relevant at the top, docking strives to order poses according to their quality with the highest quality at the top. Similarly, just as only the first page of documents is generally of interest in web page ranking, only the top 10 or so docking poses are usually considered for further investigations. Machine-learned ranking has received considerable attention in recent years due to its importance to search engines, with fundamental developments spurred on by initiatives such as the 2009 Internet Mathematics contest, the $30,000 Yahoo! Learning to Rank Challenge [143] and the ICDM 2013 Expedia Challenge. We believe that such approaches, with loss functions based on measures such as the discounted cumulative gain, and constructed and validated with completely blind features selected within an outer leave-complex-out cross-validation wrapper, will considerably improve our future ability to identify correctly docked structures.

## Methods

### Generating and evaluating the docking decoys

Ideally, all the scoring functions would be evaluated on different docking decoy sets generated using a number of different methods. This would allow the evaluation of not just scoring functions, but of whole docking protocols/scoring function combinations [124]. However, in order for the calculations to remain tractable, the consideration of increasing numbers of decoys would place a limit on the number of scoring functions which could be evaluated. For this reason, we have chosen to limit our evaluation to a large number of functions using a single decoy set generated using SwarmDock [107,108], a flexible protein-protein docking algorithm which has shown one of the top predictive performances in CAPRI [144]. SwarmDock uses normal modes to model conformational changes. It locates minima on the energy landscape using a hybrid global/local search algorithm, in which translational, orientational and normal coordinate space is simultaneously optimised. As this method produces relatively few structures, it allows us to evaluate a large number of different scoring methodologies. The decoys were generated using the SwarmDock server [109]. Briefly, non-standard residues were reverted to their precursor amino acid, missing atoms were repaired, missing residue were modelled as alanine residues, atoms were reordered to ensure agreement with the standard PDB atom ordering, and the first location was selected for atoms with multiple location indicators. The algorithm was run using default parameters [107] on a set of 118 of the 176 complexes taken from the Benchmark 4.0 [106]. Four of the Benchmark 4.0 complexes were omitted due to their large size and subsequent difficulties in evaluating a number of the scoring functions (1DE4, 1N2C, 2HMI and 2VIS). The remainder of the complexes were omitted because no solution of acceptable or better quality was generated. For each complex, around 500 decoys were generated. The decoys were scored using the 115 metrics outlined below. Some of the metrics were calculated directly from the structures. For others, the receptor, ligand and complex were evaluated separately, and the final score calculated as E_complex-(E_receptor + E_ligand). For each metric, the decoys were reranked and clustered at 3Å resolution in ascending order of energy as described previously [107,108]. For three of the metrics, NHB, SIPPER and PROPNSTS, a positive value corresponds to the most promising solutions, so these were clustered in descending order. For the ranked list of clusters, all but the first (lowest energy) member of each cluster was discarded, leaving a ranked list of structures. For these, the standard CAPRI docking quality measures were calculated: fraction of native contacts (f_nat_), interface RMSD (IRMSD) and ligand RMSD (LRMSD). These were used to classify the solutions as incorrect (f_nat_ < 0.1 or (LRMSD > 10 Å and IRMSD > 4 Å)), acceptable ((f_nat_ ≥ 0.3 and LRMSD > 5 Å and IRMSD > 2 Å) or ((f_nat_ ≥ 0.1 and f_nat_ < 0.3) and (LRMSD ≤ 10 Å or IRMSD ≤ 4 Å))), medium quality ((f_nat_ ≥ 0.5 and LRMSD > 1 Å and IRMSD > 1 Å) or ((f_nat_ ≥ 0.3 and f_nat_ < 0.5) and (LRMSD ≤ 5 Å or IRMSD ≤ 2 Å))) or high quality (f_nat_ ≥ 0.5 and (LRMSD ≤ 1 Å or IRMSD ≤ 1 Å)), in ascending order of accuracy, in accordance with the CAPRI criteria [145].

### Methods evaluated

The scoring functions evaluated are shown in Table 1. Although often experimental, biological and evolutionary information can be used to aid in the scoring of docked poses, this is not always available and here we restrict the analysis to the scoring of global docking solutions using functions which can be calculated from structure alone. Among the functions, there are many contact and distance-dependent residue-level potentials, which are prefixed with ‘CP_’ , as well as a number of atomic and near-atomic potentials, which are prefixed with ‘AP_’. A number of molecular mechanics terms were included, as well as terms obtained from docking programs and other software. Where scores are composed of multiple terms (RosettaDock, FireDock, ZRANK, ZRANK2, SIPPER, PyDock and ATTRACT), the program provided by the authors was used to calculate and weight the terms using the correct weighting scheme. Most of the functions can be either easily reprogrammed from publically available data, or can be accessed from freely available and well documented programs.

**Table 1:** A summary of the scoring functions evaluated

CP_DECK [72]	r	The DECK potential, reimplemented based on the original source code.
CP_RMFCA [146]	r	An α-carbon potential.
CP_RMFCEN1 [147]	r	A 6 bin distance-dependent centroid-centroid potential.
CP_RMFCEN2 [147]	r	A 7 bin distance-dependent centroid-centroid potential.
CP_SKOIP [111]	r	A statistical intermolecular contact potential.
CP_TB [75]	r	A docking contact potential.
CP_TSC [74]	r	A 2 bin docking potential.
PAIR [69]	p	Residue potentials that have been factorised into different energetic contributions (E_pair, E_local, E_ZS3DC, E_3DC and E_3D respectively). These are prefixed with either ‘CP_E’ for energies or ‘CP_Z’ for z-scores, and suffixed with ‘_CB’ for the β-carbon potential and ‘_MIN’ for the minimum inter-residue distance potential. The combination of these into the MixRank ranking strategy is also included. For this method, the 5 largest complexes failed to produce scores and are thus omitted.
LOCAL[69]	p	
S3DC [69]	p	
3DC [69]	p	
3D [69]	p	
CP_MIXRANK [69]	p	
CP_DDGrw [76]	r	The weighted intermolecular contact potential extracted from ΔΔG data, a preliminary model.
CP_DDGru [76]	r	The unweighted intermolecular contact potential extracted from ΔΔG data, a preliminary model.
CP_BFVK [148]	r	A number of residue-level contact potentials which have been used for protein folding studies. For these, the naming scheme and descriptions can be found elsewhere [123,149]. Contact energy matrices were downloaded from the Potentials’R’Us server.
CP_BL [150]	r	
CP_BT [151]	r	
CP_GKS [152]	r	
CP_HLPL [153]	r	
CP_MJ1 [154]	r	
CP_MJ2 [155]	r	
CP_MJ2h [155]	r	
CP_MJ3h [114]	r	
CP_MJPL [153]	r	
CP_MS [156]	r	
CP_MSBM [157,158]	r	
CP_Qa [159]	r	
CP_Qm [159]	r	
CP_Qp [159]	r	
CP_RO [160]	r	
CP_SJKG [161]	r	
CP_SKOa [162]	r	
CP_SKOb [162]	r	
CP_TD [163]	r	
CP_TEl [164]	r	
CP_TEs [164]	r	
CP_TS [165]	r	
CP_VD [166]	r	
AP_DCOMPLEX [105]	r	The DComplex potential, reimplementation based on original data file.
AP_dDFIRE [167]	d	The dDFIRE potential.
AP_DFIRE2 [168]	d	The DFIRE 2.0 potential.
AP_T1 [74]	r	The first of two two-step docking potentials.
AP_T2 [74]	r	The second of two two-step docking potentials.
AP_DOPE [169]	r	The standard DOPE potential.
AP_DOPE_HR [169]	r	The high-resolution potentials implemented in MODELLER [170,171].
AP_ACE [172]	d	The atomic contact energy desolvation score, calculated using FireDock [59].
AP_OPUS_PSP [113]	d	The OPUS_PSP folding potential.
AP_GEOMETRIC	d	The geometric potential reported in Li and Liang: Geometric packing potential function for model selection in protein structure and protein-protein binding predictions, unpublished.
AP_DARS [73]	r	The DARS decoys-as-reference-state statistical potential.
AP_URS [73]	r	The URS statistical potential.
AP_MPS [73]	r	The MFP statistical potential.
AP_WENG [173]	r	An atomic contact potential.
AP_calRW [174]	d	The distance-dependent calRW potential.
AP_calRWp [174]	d	The orientation-dependent calRWplus potential.
AP_GOAP_ALL [175]	d	The GOAP potential and its two constituent terms.
AP_GOAP_DF [175]	d	
AP_GOAP_G [175]	d	
AP_PISA [110]	d	The PISA score.
AP_DDGrw [76]	r	The weighted intermolecular contact potential extracted from ΔΔG data.
AP_DDGru [76]	r	The unweighted intermolecular contact potential extracted from ΔΔG data.
ATTRACT [61]	d	The ATTRACT scoring function, as calculated in PTools [176].
PYDOCK_TOT [54]	i	The PyDock scoring function and the electrostatics, van der Waals and desolvation terms it is composed from.
ELE [54]	i	
VDW [54]	i	
DESOLV [177]	i	
FIREDOCK [59]	d	The general purpose, enzyme-inhibitor and antibody-antigen FireDock scores and the insideness concavity score and hydrogen-bonding, π-π, cation-π and aliphatic potentials they are composed from.
FIREDOCK_EI [59]	d	
FIREDOCK_AB [59]	d	
INSIDE [59]	d	
HBOND [59]	d	
PI_PI [59]	d	
CAT_PI [59]	d	
ALIPH [59]	d	
SIPPER [67]	i	The SIPPER score and its amino-acid propensity and desolvation constituents.
PROPNSTS [67]	i	
ODA [121,122]	i	
ZRANK [57]	d	The original ZRANK scoring function.
ZRANK2 [58]	d	The reoptimised ZRANK scoring function.
NIP [79]	d	Interface packing score.
NSC [79]	d	Surface complementarity score.
ROSETTA [112]	d	The unweighted Rosetta energy, calculated using PyRosetta.
ROSETTADOCK [112]	d	The optimised RosettaDock energy, calculated using PyRosetta.
CG_PP [112]	d	The coarse-grain PyRosetta pair-potential, van der Waals, environment potential and β-potential.
CG_VDW [112]	d	
CG_ENV [112]	d	
CG_BETA [112]	d	
HBOND2 [112]	d	The atomic-resolution PyRosetta hydrogen bonding potential, amino-acid propensity scores, attractive and repulsive van der Waals energies, pair potential and desolvation energy.
AA_PROP [112]	d	
FA_ATR [112]	d	
FA_REP [112]	d	
PA_PP [112]	d	
LK_SOLV [178]	d	
NHB [112]	d	The total number of hydrogen bonds, calculated using PyRosetta.
CHARMM_TOT [179]	d	The total CHARMM energy, electrostatic energy, SASA energy and van der Waals, as calculated using the enerCHARMM script in the MMTSB toolset.
CHARMM_ELE [179]	d	
CHARMM_SASA [179]	d	
CHARMM_VDW [179]	d	
SPIDER [78]	d	The sub-graph mining based SPIDER score. As the SPIDER program only allowed scoring using a fixed receptor molecule, the unbound receptor conformation was used for this method, with a relaxed parameter set (dRMSD_CutOff = 1.0, intrCvrAbs_CutOff = 20, intrCvrPer_CutOff = 0.3, intrNumPat_CutOff = 10 and intrAveOcc_CutOff = 2).

Shown are the name of the scoring function and reference, how it was calculated (r for reimplemented, d for downloaded, p for personal communication, i for in-house), and a description/notes.

## Competing interests

The authors declare that they have no competing interests.

## Authors’ contributions

IM performed the initial conception, scoring, ranking and analysis, and drafted the manuscript. MT performed the docking calculations and classification of the docked poses. IM, MT, PAB and JFR participated in further developing the conception of the work, interpreting the data, performing critical revisions, and have read and approved the final manuscript.

## Supplementary Material

Additional file 1 Supplementary Information: This document contains additional figures and tables regarding the success rates and conditional probabilities for each scoring function, and cardinalities for each scoring function pair. Click here for file
