# Supplementary material for: The scoring of poses in protein-protein docking: current capabilities and future directions
Source: BMC Bioinformatics. 2013 Oct 1;14:286. doi: 10.1186/1471-2105-14-286 (PMC3850738; doi:10.1186/1471-2105-14-286)
Supplement: Additional file 1 — Supplementary Information: This document contains additional figures and tables regarding the success rates and conditional probabilities for each scoring function, and cardinalities for each scoring function pair. [file 1471-2105-14-286-S1.pdf]

Supplementary Material

Supplementary Figure 1. The success rates for the highest performing scoring functions. The number of complexes for which an acceptable or better solution could be found in the top 1, top 10 and top 100 solutions was calculated for each scoring function, and the best 40 scoring functions for each measure were selected. Acceptable quality solutions are shown in yellow, medium quality solutions in orange, and high quality solutions in red for the three measures (top 1 left, top 10 middle, top 100 right). The complexes are ordered by top 1 success rate.

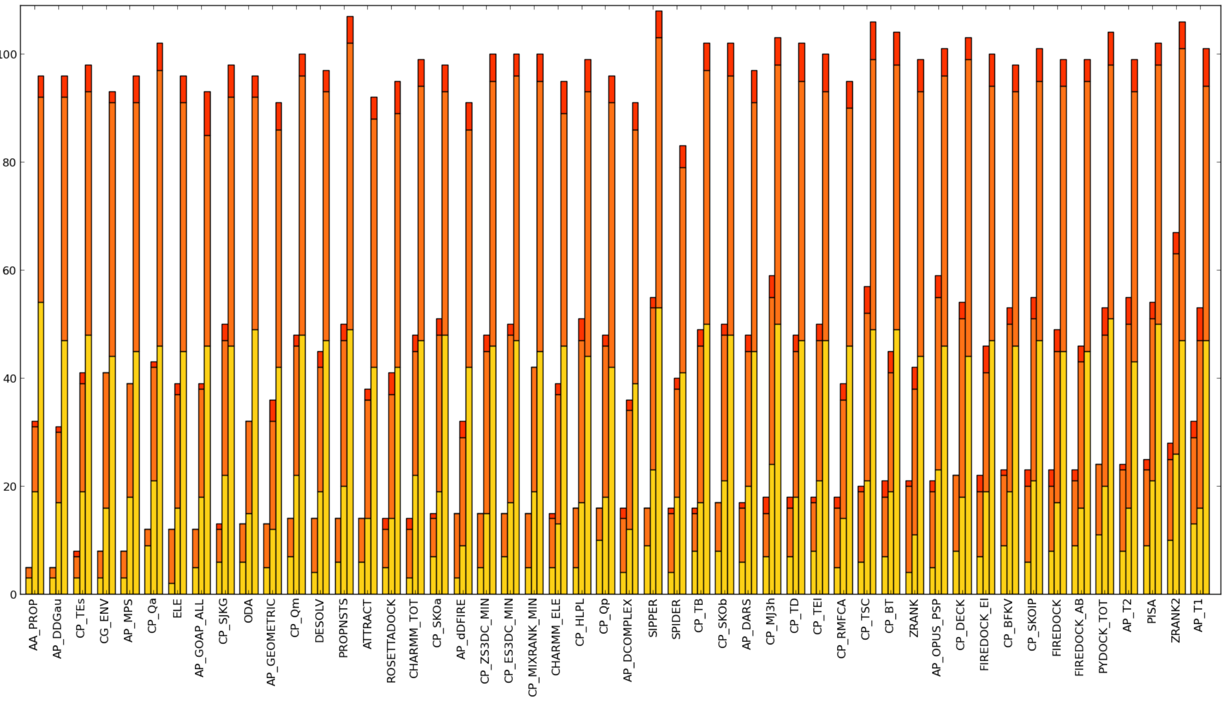

Supplementary Figure 2. The success rates for the highest performing scoring functions. The number of complexes for which an acceptable or better solution could be found in the top 1, top 10 and top 100 solutions was calculated for each scoring function, and the best 40 scoring functions for each measure were selected. Acceptable quality solutions are shown in yellow, medium quality solutions in orange, and high quality solutions in red for the three measures (top 1 left, top 10 middle, top 100 right). The complexes are ordered by top 100 success rate.

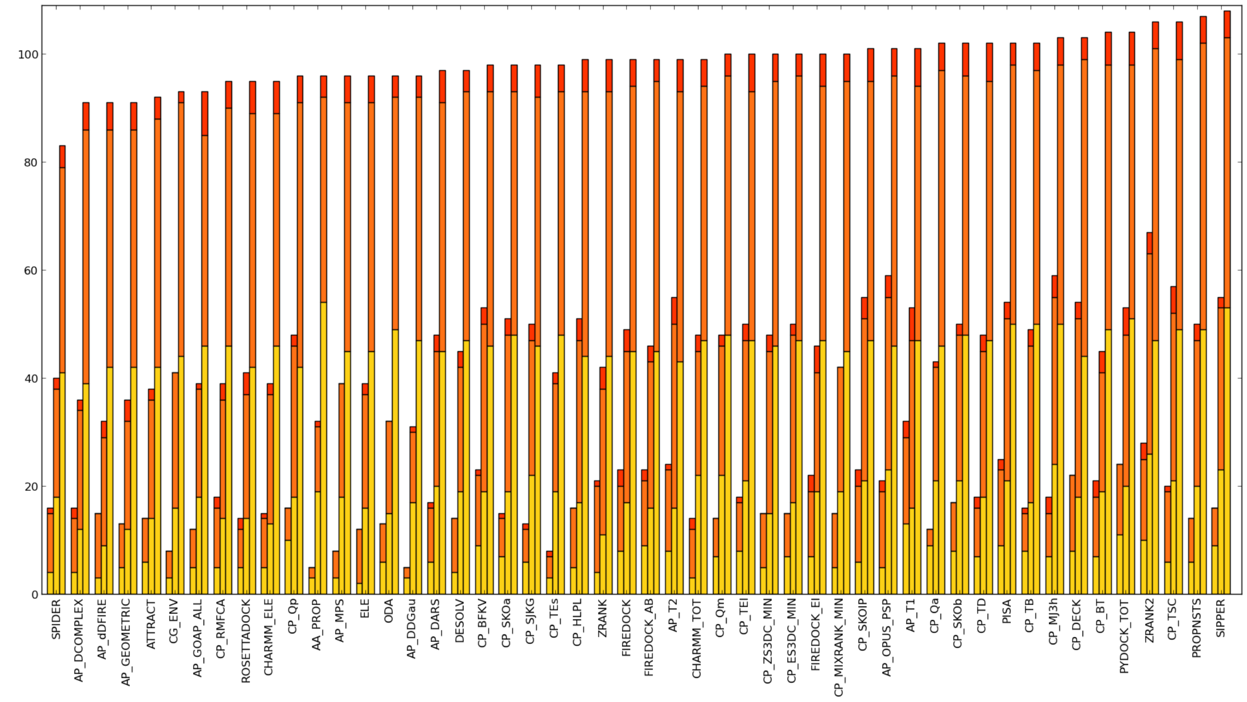

Supplementary Figure 3. The success rates for the highest performing scoring functions applied to the rigid complexes. The number of complexes for which an acceptable or better solution could be found in the top 1, top 10 and top 100 solutions was calculated for each scoring function, and the best 40 scoring functions for each measure were selected. Acceptable quality solutions are shown in yellow, medium quality solutions in orange, and high quality solutions in red for the three measures (top 1 left, top 10 middle, top 100 right). The complexes are ordered by top 1 success rate.

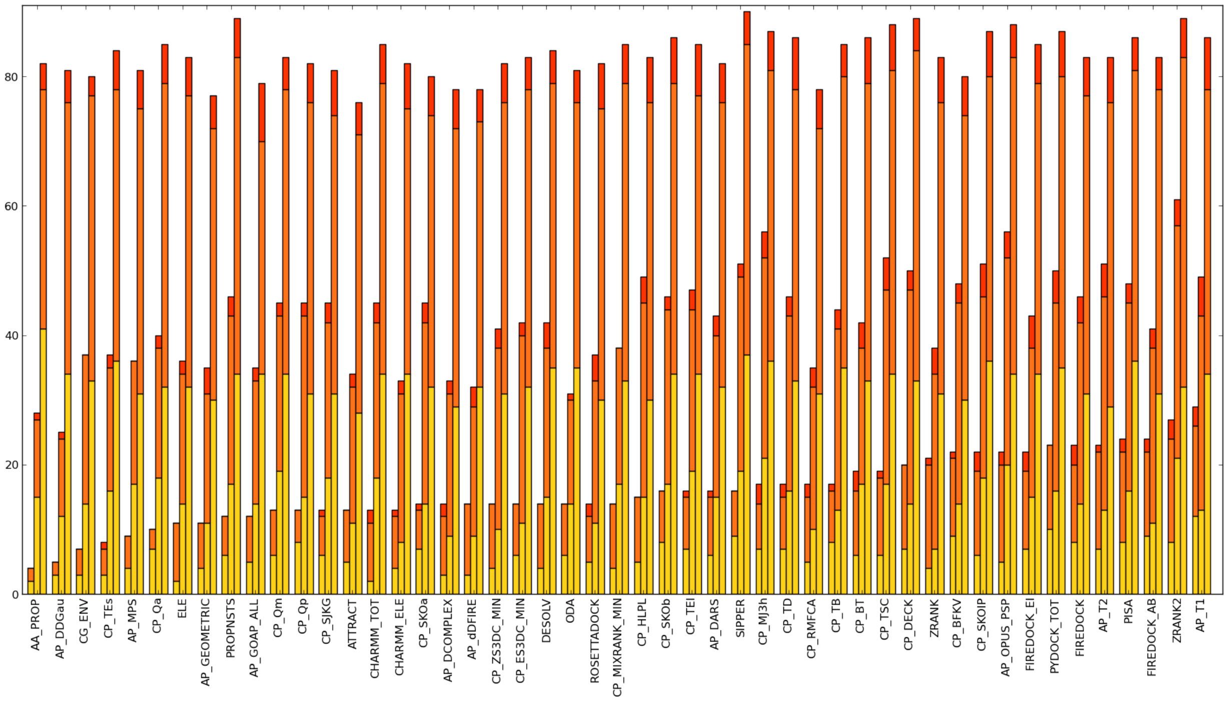

Supplementary Figure 4. The success rates for the highest performing scoring functions applied to the rigid complexes. The number of complexes for which an acceptable or better solution could be found in the top 1, top 10 and top 100 solutions was calculated for each scoring function, and the best 40 scoring functions for each measure were selected. Acceptable quality solutions are shown in yellow, medium quality solutions in orange, and high quality solutions in red for the three measures (top 1 left, top 10 middle, top 100 right). The complexes are ordered by top 10 success rate.

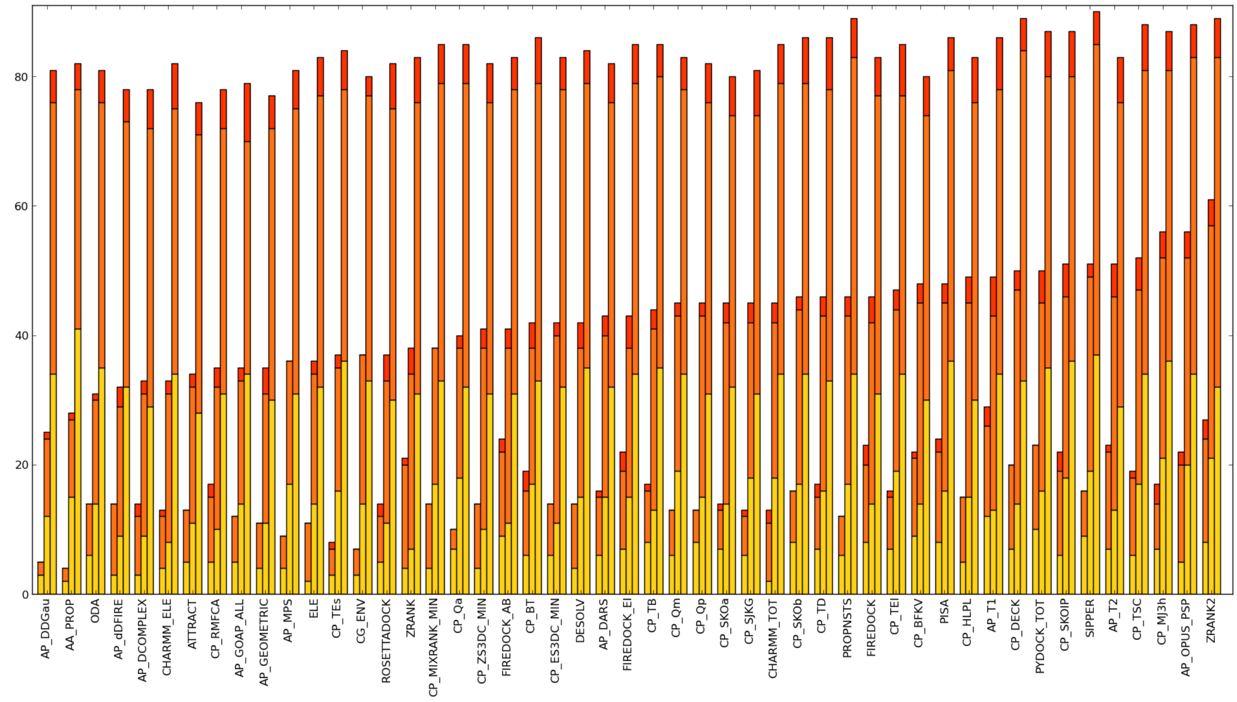

Supplementary Figure 5. The success rates for the highest performing scoring functions applied to the rigid complexes. The number of complexes for which an acceptable or better solution could be found in the top 1, top 10 and top 100 solutions was calculated for each scoring function, and the best 40 scoring functions for each measure were selected. Acceptable quality solutions are shown in yellow, medium quality solutions in orange, and high quality solutions in red for the three measures (top 1 left, top 10 middle, top 100 right). The complexes are ordered by top 100 success rate.

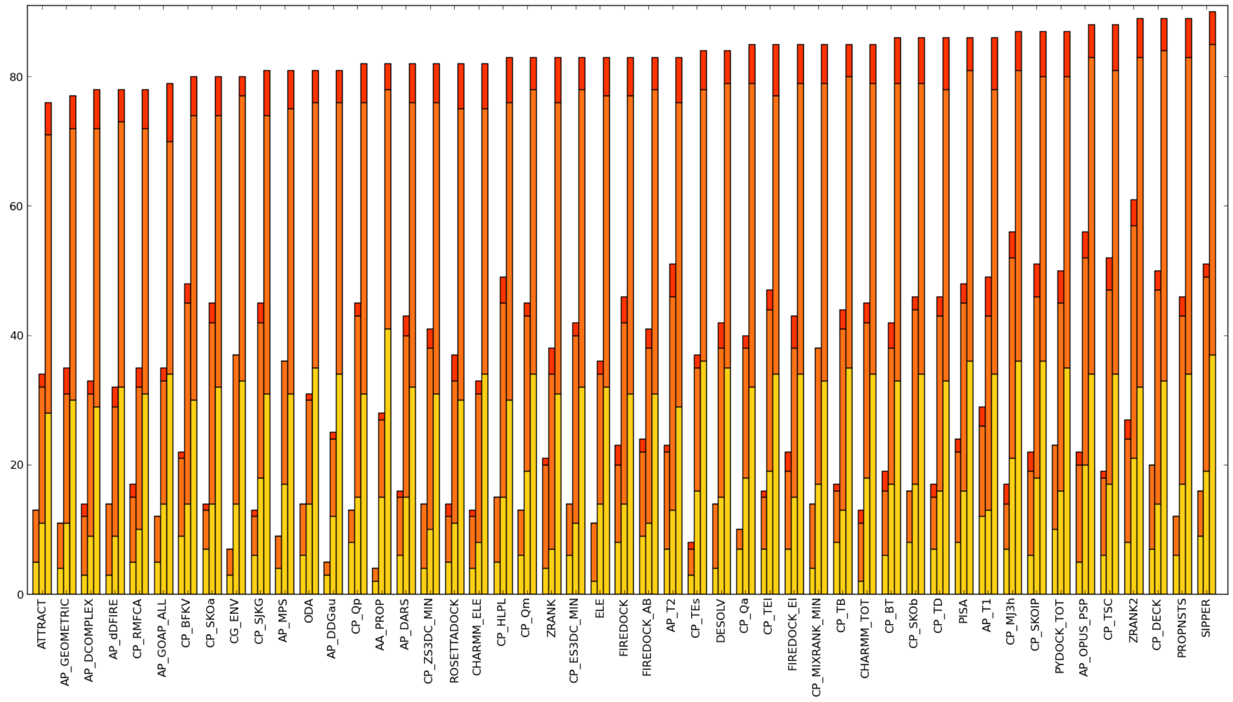

Supplementary Figure 6. The success rates for the highest performing scoring functions applied to the flexible complexes. The number of complexes for which an acceptable or better solution could be found in the top 1, top 10 and top 100 solutions was calculated for each scoring function, and the best 40 scoring functions for each measure were selected. Acceptable quality solutions are shown in yellow, medium quality solutions in orange, and high quality solutions in red for the three measures (top 1 left, top 10 middle, top 100 right). The complexes are ordered by top 1 success rate.

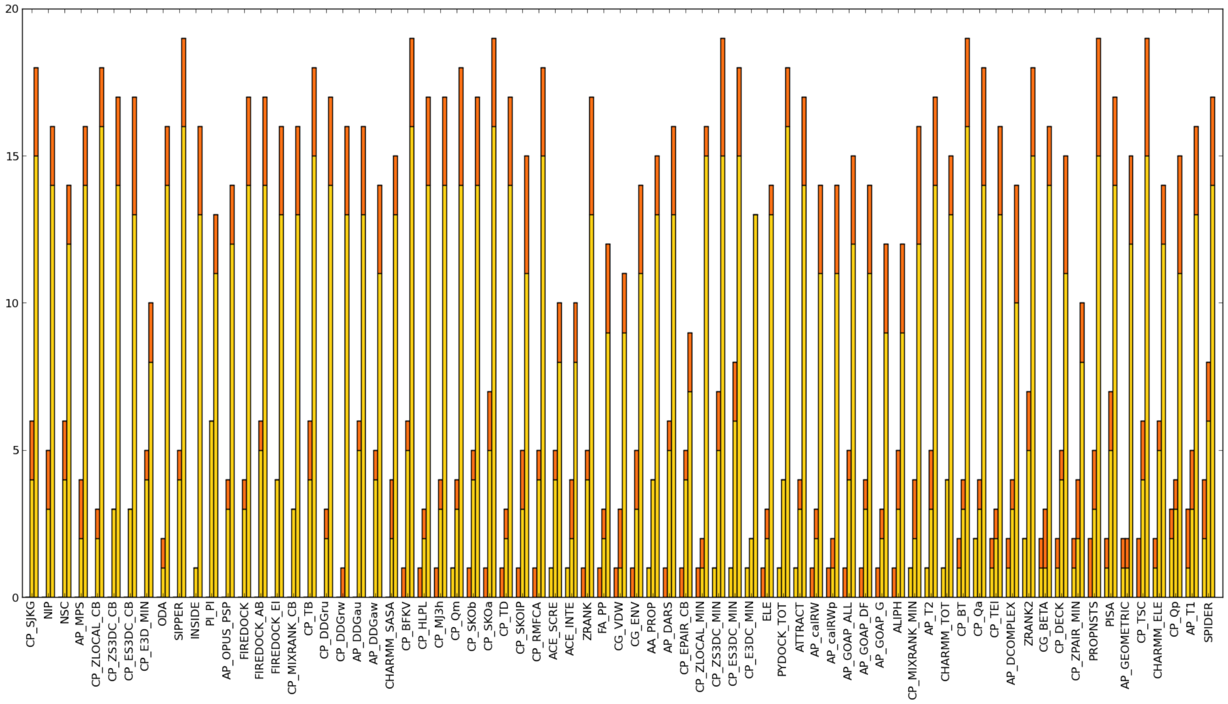

Supplementary Figure 7. The success rates for the highest performing scoring functions applied to the flexible complexes. The number of complexes for which an acceptable or better solution could be found in the top 1, top 10 and top 100 solutions was calculated for each scoring function, and the best 40 scoring functions for each measure were selected. Acceptable quality solutions are shown in yellow, medium quality solutions in orange, and high quality solutions in red for the three measures (top 1 left, top 10 middle, top 100 right). The complexes are ordered by top 10 success rate.

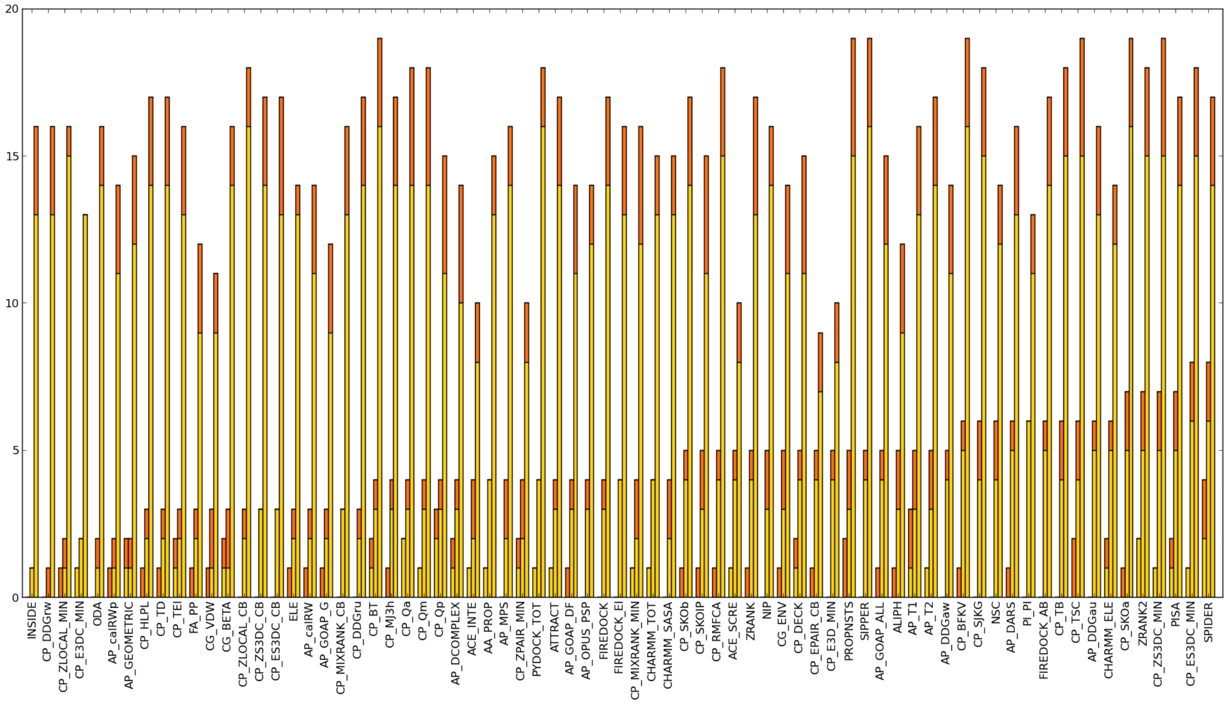

Supplementary Figure 8. The success rates for the highest performing scoring functions applied to the flexible complexes. The number of complexes for which an acceptable or better solution could be found in the top 1, top 10 and top 100 solutions was calculated for each scoring function, and the best 40 scoring functions for each measure were selected. Acceptable quality solutions are shown in yellow, medium quality solutions in orange, and high quality solutions in red for the three measures (top 1 left, top 10 middle, top 100 right). The complexes are ordered by top 100 success rate.

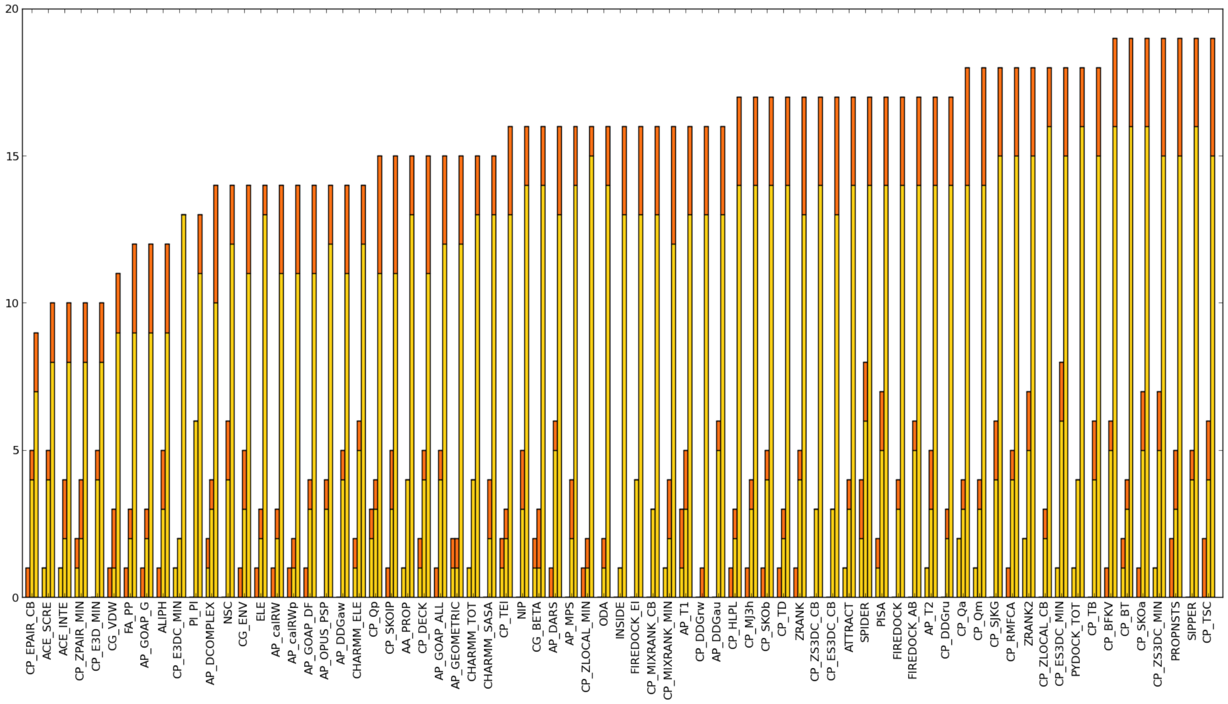

Supplementary Table 1. The number of complexes for which acceptable quality or better, medium quality or better, and high quality solutions, were found within the top 1, top 10 and top 100, for each scoring function evaluated.

Method	Acceptable			Medium			High		
	Top 1	Top 10	Top 100	Top 1	Top 10	Top 100	Top 1	Top 10	Top 100
CP_BFKV	23	54	99	14	35	53	1	3	6
CP_BL	4	20	80	3	11	42	0	0	4
CP_BT	21	46	105	14	26	56	3	4	7
CP_GKS	2	8	75	2	4	40	0	0	4
CP_HLPL	16	52	100	11	35	56	0	4	7
CP_MJPL	5	20	81	3	17	44	0	2	4
CP_MJ3h	18	60	104	11	36	54	3	4	6
CP_MJ2h	11	35	92	6	24	45	0	4	4
CP_MJ1	1	16	69	0	4	33	0	0	4
CP_MJ2	3	16	80	2	7	40	0	1	4
CP_MSBM	3	19	78	2	10	39	0	0	3
CP_MS	4	23	81	3	8	44	0	1	5
CP_Qa	12	44	103	3	23	57	0	2	6
CP_Qm	14	49	101	7	27	53	0	2	5
CP_Qp	16	49	97	6	31	55	0	2	6
CP_RO	5	29	90	2	15	44	1	2	5
CP_SKOb	17	51	103	9	30	55	0	2	7
CP_SKOa	15	52	99	8	33	51	1	3	6
CP_SJKG	13	51	99	7	29	53	1	3	7
CP_TD	18	49	103	11	31	56	2	3	8
CP_TEl	18	50	101	10	29	54	1	3	8
CP_TEs	8	41	99	5	22	51	1	2	6
CP_TS	7	29	82	3	19	45	0	1	5
CP_VD	7	37	93	5	25	50	0	2	5
CP_SKOIP	23	56	102	17	35	55	3	5	7
AP_DCOMPLEX	16	37	92	12	25	53	2	2	6
AP_dDFIRE	15	33	92	12	24	50	0	3	5
AP_DFIRE2	10	27	92	7	21	47	0	3	5
CP_RMFCEN1	10	32	93	5	22	49	1	3	4
CP_RMFCEN2	9	33	91	5	21	50	0	3	5
CP_RMFCA	18	40	96	13	26	50	2	3	6
AP_DOPE	7	19	83	6	9	44	0	0	7
AP_DOPE_HR	7	24	89	5	15	44	0	0	5
ZRANK	22	43	100	18	32	56	1	4	7
ZRANK2	29	68	107	19	42	60	3	4	6
ROT_S	4	18	67	1	9	32	0	0	2
TRANS_S	4	19	81	3	9	44	1	1	6
NIP	3	28	76	1	12	39	0	0	3
NSC	9	37	85	5	18	39	0	0	3
FA_ATR	3	29	82	2	19	44	0	2	4
FA_REP	4	19	87	2	10	45	0	0	4
LK_SOLV	4	19	75	1	6	30	0	1	4
FA_PP	4	15	86	2	8	48	0	0	4
CG_VDW	2	25	82	1	14	45	0	1	5
CG_PP	1	14	72	1	5	42	0	0	6
CG_ENV	8	42	94	5	25	50	0	0	3
CG_BETA	2	16	77	1	10	35	0	0	2
HBOND2	5	23	79	3	15	44	1	3	6
ROSETTA	6	17	84	2	6	42	0	0	6
AA_PROP	5	32	97	2	13	43	0	1	4
AP_DARS	17	49	98	11	29	53	1	3	6
AP_URS	8	25	84	8	18	43	1	2	5
AP_MPS	9	40	97	5	21	52	0	0	6
AP_WENG	3	14	81	2	5	39	1	1	6
CP_DECK	22	55	104	14	37	60	0	3	5
CP_ZPAIR_CB	1	10	63	1	5	29	0	1	5
CP_ZLOCAL_CB	2	15	81	1	8	39	0	0	3
CP_ZS3DC_CB	4	22	92	3	12	48	0	1	5
CP_Z3DC_CB	0	7	58	0	2	27	0	0	3
CP_EPAIR_CB	4	16	75	3	6	37	1	1	4
CP_ELOCAL_CB	4	21	71	2	15	34	0	0	3
CP_ES3DC_CB	6	21	92	3	12	45	0	1	3
CP_E3DC_CB	1	8	59	1	4	25	0	0	1

Table continued on next page

Table continued from previous page

Method	Acceptable			Medium			High		
	Top 1	Top 10	Top 100	Top 1	Top 10	Top 100	Top 1	Top 10	Top 100
CP_E3D_CB	4	21	69	2	12	35	0	0	4
CP_ZPAIR_MIN	3	23	81	1	10	44	0	1	6
CP_ZLOCAL_MIN	1	13	74	1	7	35	0	0	2
CP_ZS3DC_MIN	15	48	101	10	33	55	0	3	6
CP_Z3DC_MIN	0	13	72	0	3	27	0	1	1
CP_EPAIR_MIN	3	25	80	2	10	41	0	2	6
CP_ELOCAL_MIN	6	24	75	4	11	39	0	0	3
CP_ES3DC_MIN	15	50	101	8	33	54	0	2	5
CP_E3DC_MIN	4	18	76	1	7	35	0	0	1
CP_E3D_MIN	7	28	77	5	14	43	0	0	5
ELE	12	39	97	10	23	52	0	2	6
DESOLV	14	46	98	10	27	51	0	4	5
VDW	6	26	81	4	18	42	0	1	4
PYDOCK_TOT	24	54	105	13	34	54	0	5	7
ODA	14	33	97	8	18	48	0	1	5
PROPNSTS	14	51	108	8	31	59	0	3	6
SIPPER	16	56	109	7	33	56	0	2	5
ATTRACT	14	38	93	8	24	51	0	2	5
SPIDER	16	41	84	12	22	43	1	2	5
PISA	26	55	103	17	34	53	2	3	5
ROSETTADOCK	14	41	96	9	27	54	2	4	7
AP_calRW	9	29	89	7	22	47	0	3	5
AP_calRWp	10	29	90	7	22	47	0	3	5
AP_GOAP_ALL	13	40	94	8	22	48	0	2	9
AP_GOAP_DF	9	31	90	7	22	47	0	2	5
AP_GOAP_G	8	25	84	5	13	46	0	2	8
AP_ACE	4	34	81	3	18	44	0	4	7
INSIDE	9	26	92	6	17	45	1	1	6
HBOND	7	23	80	4	16	47	1	2	5
PI_PI	6	27	92	3	13	53	0	0	4
CAT_PI	6	21	90	2	9	48	0	1	8
ALIPH	9	32	82	7	22	45	2	3	4
AP_OPUS_PSP	22	60	102	17	37	56	2	4	5
AP_GEOMETRIC	13	37	92	8	25	50	0	4	5
FIREDOCK	23	50	100	15	33	55	3	4	6
FIREDOCK_AB	24	47	100	15	31	55	2	3	5
FIREDOCK_EI	22	47	101	15	28	54	3	5	6
CP_MIXRANK_CB	4	18	85	3	8	40	0	1	4
CP_MIXRANK_MIN	15	42	101	10	23	56	0	0	6
CP_TB	17	50	103	9	33	53	1	3	5
CP_TSC	21	58	107	15	37	58	1	5	7
AP_T1	32	54	102	19	38	55	3	6	8
AP_T2	24	56	100	16	40	57	1	5	7
NHB	4	24	91	3	15	49	0	3	7
CP_DDGru	7	29	93	4	15	48	0	0	7
CP_DDGrw	5	30	91	3	21	46	0	1	4
AP_DDGau	5	31	97	2	14	50	0	1	5
AP_DDGaw	10	37	94	9	22	54	1	3	7
CHARMM_TOT	14	49	100	11	27	53	2	3	6
CHARMM_ELE	15	39	96	10	26	50	1	2	7
CHARMM_SASA	2	27	78	2	14	39	0	1	3
CHARMM_VDW	6	23	77	4	14	41	0	0	3

Supplementary Table 2. Conditional probibilities of finding an acceptable solution within the top 10 or top 100, given the existence of an acceptable or better solution, or a medium or better solution. Probabilities are shown as percentages.

Method	Top 10			Top 100		
	Acceptable	Medium	Difference	Acceptable	Medium	Difference
CP_MJ1	13.01	5.41	-7.21	56.10	44.59	-6.09
CP_MS	18.70	10.81	-6.79	65.85	59.46	1.20
LK_SOLV	15.45	8.11	-6.58	60.98	40.54	-15.94
AA_PROP	26.02	17.57	-6.48	78.86	58.11	-13.95
CP_Z3DC_MIN	10.57	4.05	-6.26	58.54	36.49	-18.16
CP_EPAIR_MIN	20.33	13.51	-5.31	65.04	55.41	-2.72
ROSETTA	13.82	8.11	-4.88	68.29	56.76	-4.52
NIP	22.76	16.22	-4.68	61.79	52.70	-2.50
CP_E3DC_MIN	14.63	9.46	-4.14	61.79	47.30	-8.85
AP_DDGau	25.20	18.92	-4.05	78.86	67.57	-2.84
CP_EPAIR_CB	13.01	8.11	-4.04	60.98	50.00	-4.83
AP_WENG	11.38	6.76	-3.93	65.85	52.70	-6.74
CG_PP	11.38	6.76	-3.93	58.54	56.76	5.65
CP_ZPAIR_MIN	18.70	13.51	-3.62	65.85	59.46	1.20
CAT_PI	17.07	12.16	-3.51	73.17	64.86	-0.08
CP_ELOCAL_MIN	19.51	14.86	-2.88	60.98	52.70	-1.65
NSC	30.08	24.32	-2.78	69.11	52.70	-10.13
CP_Z3DC_CB	5.69	2.70	-2.76	47.15	36.49	-6.30
CP_MIXRANK_CB	14.63	10.81	-2.56	69.11	54.05	-8.54
CP_MJ2	13.01	9.46	-2.45	65.04	54.05	-4.30
PI_PI	21.95	17.57	-2.25	74.80	71.62	6.16
AP_DOPE	15.45	12.16	-1.82	67.48	59.46	-0.50
TRANS_S	15.45	12.16	-1.82	65.85	59.46	1.20
CP_E3D_MIN	22.76	18.92	-1.51	62.60	58.11	3.00
ROT_S	14.63	12.16	-0.97	54.47	43.24	-5.99
CP_Qa	35.77	31.08	-0.78	83.74	77.03	3.19
CP_DDGru	23.58	20.27	-0.77	75.61	64.86	-2.62
CP_RO	23.58	20.27	-0.77	73.17	59.46	-6.43
CHARMM_SASA	21.95	18.92	-0.66	63.41	52.70	-4.20
AP_MPS	32.52	28.38	-0.56	78.86	70.27	0.34
AP_GOAP_G	20.33	17.57	-0.55	68.29	62.16	1.83
CP_ZPAIR_CB	8.13	6.76	-0.54	51.22	39.19	-7.36
CP_E3DC_CB	6.50	5.41	-0.43	47.97	33.78	-10.32
CP_GKS	6.50	5.41	-0.43	60.98	54.05	-0.07
AP_ACE	27.64	24.32	-0.24	65.85	59.46	1.20
CP_MSBM	15.45	13.51	-0.23	63.41	52.70	-4.20
FA_REP	15.45	13.51	-0.23	70.73	60.81	-2.30
CP_ZLOCAL_CB	12.20	10.81	-0.01	65.85	52.70	-6.74
FA_PP	12.20	10.81	-0.01	69.92	64.86	3.31
CP_ZLOCAL_MIN	10.57	9.46	0.09	60.16	47.30	-7.16
CP_TEs	33.33	29.73	0.17	80.49	68.92	-2.95
SPIDER	33.33	29.73	0.17	68.29	58.11	-2.93
CP_ZS3DC_CB	17.89	16.22	0.40	74.80	64.86	-1.78
CP_BL	16.26	14.86	0.51	65.04	56.76	-1.13
ODA	26.83	24.32	0.61	78.86	64.86	-6.01
CP_MIXRANK_MIN	34.15	31.08	0.91	82.11	75.68	3.30
AP_GOAP_ALL	32.52	29.73	1.02	76.42	64.86	-3.47
CG_VDW	20.33	18.92	1.04	66.67	60.81	1.94
CP_E3D_CB	17.07	16.22	1.25	56.10	47.30	-2.92
CP_ES3DC_CB	17.07	16.22	1.25	74.80	60.81	-6.54
CHARMM_TOT	39.84	36.49	1.33	81.30	71.62	-0.62
CP_Qm	39.84	36.49	1.33	82.11	71.62	-1.47
CP_BT	37.40	35.14	2.29	85.37	75.68	-0.09
CG_BETA	13.01	13.51	2.31	62.60	47.30	-9.70
CHARMM_VDW	18.70	18.92	2.73	62.60	55.41	-0.17
CP_SJKG	41.46	39.19	2.81	80.49	71.62	0.23
ELE	31.71	31.08	3.46	78.86	70.27	0.34
AP_DOPE_HR	19.51	20.27	3.47	72.36	59.46	-5.58
NHB	19.51	20.27	3.47	73.98	66.22	0.66
AP_DDGaw	30.08	29.73	3.56	76.42	72.97	6.05
CP_TEl	40.65	39.19	3.66	82.11	72.97	0.12
DESOLV	37.40	36.49	3.87	79.67	68.92	-2.10
CG_ENV	34.15	33.78	4.09	76.42	67.57	-0.30

Table continued on next page

Table continued from previous page

Method	Top 10			Top 100		
	Acceptable	Medium	Difference	Acceptable	Medium	Difference
HBOND2	18.70	20.27	4.32	64.23	59.46	2.89
CP_SKOb	41.46	40.54	4.40	83.74	74.32	0.01
AP_DARS	39.84	39.19	4.51	79.67	71.62	1.08
FIREDOCK_EI	38.21	37.84	4.61	82.11	72.97	0.12
SIPPER	45.53	44.59	4.92	88.62	75.68	-3.48
INSIDE	21.14	22.97	4.95	74.80	60.81	-6.54
CP_RMFCEN2	26.83	28.38	5.37	73.98	67.57	2.25
CP_TS	23.58	25.68	5.58	66.67	60.81	1.94
FA_ATR	23.58	25.68	5.58	66.67	59.46	0.35
ATTRACT	30.89	32.43	5.89	75.61	68.92	2.14
HBOND	18.70	21.62	5.91	65.04	63.51	6.81
PROPNSTS	41.46	41.89	5.99	87.80	79.73	2.13
CP_ELOCAL_CB	17.07	20.27	6.01	57.72	45.95	-6.20
CP_MJ3h	48.78	48.65	6.30	84.55	72.97	-2.42
VDW	21.14	24.32	6.54	65.85	56.76	-1.98
PISA	44.72	45.95	7.36	83.74	71.62	-3.16
CP_RMFCA	32.52	35.14	7.37	78.05	67.57	-1.99
AP_URS	20.33	24.32	7.38	68.29	58.11	-2.93
CP_Qp	39.84	41.89	7.68	78.86	74.32	5.10
CP_TD	39.84	41.89	7.68	83.74	75.68	1.60
ALIPH	26.02	29.73	7.80	66.67	60.81	1.94
CP_RMFCEN1	26.02	29.73	7.80	75.61	66.22	-1.04
AP_OPUS_PSP	48.78	50.00	7.88	82.93	75.68	2.45
CP_DDGrw	24.39	28.38	7.91	73.98	62.16	-4.10
CP_SKOIP	45.53	47.30	8.10	82.93	74.32	0.86
ROSETTADOCK	33.33	36.49	8.11	78.05	72.97	4.36
PYDOCK_TOT	43.90	45.95	8.21	85.37	72.97	-3.27
CHARMM_ELE	31.71	35.14	8.22	78.05	67.57	-1.99
CP_SKOa	42.28	44.59	8.31	80.49	68.92	-2.95
AP_DCOMPLEX	30.08	33.78	8.33	74.80	71.62	6.16
AP_GEOMETRIC	30.08	33.78	8.33	74.80	67.57	1.40
CP_VD	30.08	33.78	8.33	75.61	67.57	0.55
CP_MJ2h	28.46	32.43	8.43	74.80	60.81	-6.54
AP_GOAP_DF	25.20	29.73	8.65	73.17	63.51	-1.67
ZRANK2	55.28	56.76	9.04	86.99	81.08	4.56
FIREDOCK_AB	38.21	41.89	9.38	81.30	74.32	2.56
CP_TSC	47.15	50.00	9.58	86.99	78.38	1.39
CP_BFKV	43.90	47.30	9.79	80.49	71.62	0.23
CP_ES3DC_MIN	40.65	44.59	10.01	82.11	72.97	0.12
CP_TB	40.65	44.59	10.01	83.74	71.62	-3.16
FIREDOCK	40.65	44.59	10.01	81.30	74.32	2.56
CP_MJPL	16.26	22.97	10.03	65.85	59.46	1.20
AP_dDFIRE	26.83	32.43	10.13	74.80	67.57	1.40
AP_calRW	23.58	29.73	10.34	72.36	63.51	-0.82
AP_calRWp	23.58	29.73	10.34	73.17	63.51	-1.67
AP_DFIRE2	21.95	28.38	10.45	74.80	63.51	-3.36
CP_HLPL	42.28	47.30	11.49	81.30	75.68	4.14
CP_ZS3DC_MIN	39.02	44.59	11.70	82.11	74.32	1.71
CP_DECK	44.72	50.00	12.12	84.55	81.08	7.10
ZRANK	34.96	43.24	14.35	81.30	75.68	4.14
AP_T1	43.90	51.35	14.55	82.93	74.32	0.86
AP_T2	45.53	54.05	16.03	81.30	77.03	5.73

Supplementary Table 3. A comparison of the sets of complexes for which an acceptable or better solution could be found in the top 10 structures. For each pair of scoring functions, A and B, their size ($|A|$ and $|B|$), unison ($|A \cup B|$), symmetric difference ($|A \ominus B|$) and relative complements ($|A \backslash B|$ and $|B \backslash A|$), are shown.

Method A	Method B	$\|A\|$	$\|B\|$	$\|A \cup B\|$	$\|A \ominus B\|$	$\|A \backslash B\|$	$\|B \backslash A\|$
ZRANK2	AP_T1	68	54	74	26	20	6
PISA	AP_T1	55	54	66	23	12	11
PISA	ZRANK2	55	68	72	21	4	17
AP_T2	AP_T1	56	54	64	18	10	8
AP_T2	ZRANK2	56	68	72	20	4	16
AP_T2	PISA	56	55	67	23	12	11
PYDOCK_TOT	AP_T1	54	54	69	30	15	15
PYDOCK_TOT	ZRANK2	54	68	76	30	8	22
PYDOCK_TOT	PISA	54	55	73	37	18	19
PYDOCK_TOT	AP_T2	54	56	71	32	15	17
FIREDOCK_AB	AP_T1	47	54	63	25	9	16
FIREDOCK_AB	ZRANK2	47	68	72	29	4	25
FIREDOCK_AB	PISA	47	55	63	24	8	16
FIREDOCK_AB	AP_T2	47	56	63	23	7	16
FIREDOCK_AB	PYDOCK_TOT	47	54	64	27	10	17
FIREDOCK	AP_T1	50	54	63	22	9	13
FIREDOCK	ZRANK2	50	68	74	30	6	24
FIREDOCK	PISA	50	55	67	29	12	17
FIREDOCK	AP_T2	50	56	63	20	7	13
FIREDOCK	PYDOCK_TOT	50	54	64	24	10	14
FIREDOCK	FIREDOCK_AB	50	47	53	9	6	3
CP_SKOIP	AP_T1	56	54	72	34	18	16
CP_SKOIP	ZRANK2	56	68	76	28	8	20
CP_SKOIP	PISA	56	55	73	35	18	17
CP_SKOIP	AP_T2	56	56	73	34	17	17
CP_SKOIP	PYDOCK_TOT	56	54	72	34	18	16
CP_SKOIP	FIREDOCK_AB	56	47	73	43	26	17
CP_SKOIP	FIREDOCK	56	50	72	38	22	16
CP_BFKV	AP_T1	54	54	74	40	20	20
CP_BFKV	ZRANK2	54	68	80	38	12	26
CP_BFKV	PISA	54	55	74	39	19	20
CP_BFKV	AP_T2	54	56	72	34	16	18
CP_BFKV	PYDOCK_TOT	54	54	74	40	20	20
CP_BFKV	FIREDOCK_AB	54	47	73	45	26	19
CP_BFKV	FIREDOCK	54	50	75	46	25	21
CP_BFKV	CP_SKOIP	54	56	76	42	20	22
FIREDOCK_EI	AP_T1	47	54	63	25	9	16
FIREDOCK_EI	ZRANK2	47	68	75	35	7	28
FIREDOCK_EI	PISA	47	55	64	26	9	17
FIREDOCK_EI	AP_T2	47	56	66	29	10	19
FIREDOCK_EI	PYDOCK_TOT	47	54	63	25	9	16
FIREDOCK_EI	FIREDOCK_AB	47	47	54	14	7	7
FIREDOCK_EI	FIREDOCK	47	50	54	11	4	7
FIREDOCK_EI	CP_SKOIP	47	56	69	35	13	22
FIREDOCK_EI	CP_BFKV	47	54	73	45	19	26
CP_DECK	AP_T1	55	54	72	35	18	17
CP_DECK	ZRANK2	55	68	77	31	9	22
CP_DECK	PISA	55	55	70	30	15	15
CP_DECK	AP_T2	55	56	74	37	18	19
CP_DECK	PYDOCK_TOT	55	54	73	37	19	18
CP_DECK	FIREDOCK_AB	55	47	72	42	25	17
CP_DECK	FIREDOCK	55	50	72	39	22	17
CP_DECK	CP_SKOIP	55	56	72	33	16	17
CP_DECK	CP_BFKV	55	54	76	43	22	21
CP_DECK	FIREDOCK_EI	55	47	71	40	24	16
AP_OPUS_PSP	AP_T1	60	54	69	24	15	9
AP_OPUS_PSP	ZRANK2	60	68	73	18	5	13
AP_OPUS_PSP	PISA	60	55	68	21	13	8
AP_OPUS_PSP	AP_T2	60	56	71	26	15	11
AP_OPUS_PSP	PYDOCK_TOT	60	54	74	34	20	14
AP_OPUS_PSP	FIREDOCK_AB	60	47	69	31	22	9
AP_OPUS_PSP	FIREDOCK	60	50	71	32	21	11
AP_OPUS_PSP	CP_SKOIP	60	56	73	30	17	13

Table continued on next page

Table continued from previous page

Method A	Method B	$\|A\|$	$\|B\|$	$\|A \cup B\|$	$\|A \ominus B\|$	$\|A \backslash B\|$	$\|B \backslash A\|$
AP_OPUS_PSP	CP_BFKV	60	54	77	40	23	17
AP_OPUS_PSP	FIREDOCK_EI	60	47	69	31	22	9
AP_OPUS_PSP	CP_DECK	60	55	73	31	18	13
ZRANK	AP_T1	43	54	58	19	4	15
ZRANK	ZRANK2	43	68	70	29	2	27
ZRANK	PISA	43	55	61	24	6	18
ZRANK	AP_T2	43	56	61	23	5	18
ZRANK	PYDOCK_TOT	43	54	64	31	10	21
ZRANK	FIREDOCK_AB	43	47	51	12	4	8
ZRANK	FIREDOCK	43	50	54	15	4	11
ZRANK	CP_SKOIP	43	56	72	45	16	29
ZRANK	CP_BFKV	43	54	70	43	16	27
ZRANK	FIREDOCK_EI	43	47	55	20	8	12
ZRANK	CP_DECK	43	55	71	44	16	28
ZRANK	AP_OPUS_PSP	43	60	66	29	6	23
CP_BT	AP_T1	46	54	63	26	9	17
CP_BT	ZRANK2	46	68	75	36	7	29
CP_BT	PISA	46	55	67	33	12	21
CP_BT	AP_T2	46	56	65	28	9	19
CP_BT	PYDOCK_TOT	46	54	68	36	14	22
CP_BT	FIREDOCK_AB	46	47	62	31	15	16
CP_BT	FIREDOCK	46	50	65	34	15	19
CP_BT	CP_SKOIP	46	56	73	44	17	27
CP_BT	CP_BFKV	46	54	60	20	6	14
CP_BT	FIREDOCK_EI	46	47	64	35	17	18
CP_BT	CP_DECK	46	55	70	39	15	24
CP_BT	AP_OPUS_PSP	46	60	69	32	9	23
CP_BT	ZRANK	46	43	60	31	17	14
CP_TSC	AP_T1	58	54	69	26	15	11
CP_TSC	ZRANK2	58	68	73	20	5	15
CP_TSC	PISA	58	55	71	29	16	13
CP_TSC	AP_T2	58	56	69	24	13	11
CP_TSC	PYDOCK_TOT	58	54	75	38	21	17
CP_TSC	FIREDOCK_AB	58	47	69	33	22	11
CP_TSC	FIREDOCK	58	50	72	36	22	14
CP_TSC	CP_SKOIP	58	56	76	38	20	18
CP_TSC	CP_BFKV	58	54	76	40	22	18
CP_TSC	FIREDOCK_EI	58	47	74	43	27	16
CP_TSC	CP_DECK	58	55	75	37	20	17
CP_TSC	AP_OPUS_PSP	58	60	72	26	12	14
CP_TSC	ZRANK	58	43	66	31	23	8
CP_TSC	CP_BT	58	46	68	32	22	10
CP_RMFCA	AP_T1	40	54	60	26	6	20
CP_RMFCA	ZRANK2	40	68	70	32	2	30
CP_RMFCA	PISA	40	55	60	25	5	20
CP_RMFCA	AP_T2	40	56	61	26	5	21
CP_RMFCA	PYDOCK_TOT	40	54	60	26	6	20
CP_RMFCA	FIREDOCK_AB	40	47	55	23	8	15
CP_RMFCA	FIREDOCK	40	50	59	28	9	19
CP_RMFCA	CP_SKOIP	40	56	66	36	10	26
CP_RMFCA	CP_BFKV	40	54	65	36	11	25
CP_RMFCA	FIREDOCK_EI	40	47	56	25	9	16
CP_RMFCA	CP_DECK	40	55	68	41	13	28
CP_RMFCA	AP_OPUS_PSP	40	60	65	30	5	25
CP_RMFCA	ZRANK	40	43	51	19	8	11
CP_RMFCA	CP_BT	40	46	57	28	11	17
CP_RMFCA	CP_TSC	40	58	64	30	6	24
CP_TEl	AP_T1	50	54	70	36	16	20
CP_TEl	ZRANK2	50	68	75	32	7	25
CP_TEl	PISA	50	55	72	39	17	22
CP_TEl	AP_T2	50	56	71	36	15	21
CP_TEl	PYDOCK_TOT	50	54	70	36	16	20
CP_TEl	FIREDOCK_AB	50	47	68	39	21	18
CP_TEl	FIREDOCK	50	50	70	40	20	20
CP_TEl	CP_SKOIP	50	56	72	38	16	22
CP_TEl	CP_BFKV	50	54	67	30	13	17
CP_TEl	FIREDOCK_EI	50	47	68	39	21	18
CP_TEl	CP_DECK	50	55	71	37	16	21

Table continued on next page

Table continued from previous page

Method A	Method B	$\|A\|$	$\|B\|$	$\|A \cup B\|$	$\|A \ominus B\|$	$\|A \backslash B\|$	$\|B \backslash A\|$
CP_TEl	AP_OPUS_PSP	50	60	75	40	15	25
CP_TEl	ZRANK	50	43	66	39	23	16
CP_TEl	CP_BT	50	46	62	28	16	12
CP_TEl	CP_TSC	50	58	70	32	12	20
CP_TEl	CP_RMFCA	50	40	64	38	24	14
CP_TD	AP_T1	49	54	68	33	14	19
CP_TD	ZRANK2	49	68	74	31	6	25
CP_TD	PISA	49	55	68	32	13	19
CP_TD	AP_T2	49	56	70	35	14	21
CP_TD	PYDOCK_TOT	49	54	71	39	17	22
CP_TD	FIREDOCK_AB	49	47	65	34	18	16
CP_TD	FIREDOCK	49	50	67	35	17	18
CP_TD	CP_SKOIP	49	56	69	33	13	20
CP_TD	CP_BFKV	49	54	68	33	14	19
CP_TD	FIREDOCK_EI	49	47	65	34	18	16
CP_TD	CP_DECK	49	55	65	26	10	16
CP_TD	AP_OPUS_PSP	49	60	70	31	10	21
CP_TD	ZRANK	49	43	65	38	22	16
CP_TD	CP_BT	49	46	60	25	14	11
CP_TD	CP_TSC	49	58	70	33	12	21
CP_TD	CP_RMFCA	49	40	63	37	23	14
CP_TD	CP_TEl	49	50	64	29	14	15
CP_MJ3h	AP_T1	60	54	71	28	17	11
CP_MJ3h	ZRANK2	60	68	78	28	10	18
CP_MJ3h	PISA	60	55	74	33	19	14
CP_MJ3h	AP_T2	60	56	73	30	17	13
CP_MJ3h	PYDOCK_TOT	60	54	70	26	16	10
CP_MJ3h	FIREDOCK_AB	60	47	70	33	23	10
CP_MJ3h	FIREDOCK	60	50	69	28	19	9
CP_MJ3h	CP_SKOIP	60	56	74	32	18	14
CP_MJ3h	CP_BFKV	60	54	72	30	18	12
CP_MJ3h	FIREDOCK_EI	60	47	68	29	21	8
CP_MJ3h	CP_DECK	60	55	75	35	20	15
CP_MJ3h	AP_OPUS_PSP	60	60	73	26	13	13
CP_MJ3h	ZRANK	60	43	69	35	26	9
CP_MJ3h	CP_BT	60	46	65	24	19	5
CP_MJ3h	CP_TSC	60	58	75	32	17	15
CP_MJ3h	CP_RMFCA	60	40	68	36	28	8
CP_MJ3h	CP_TEl	60	50	66	22	16	6
CP_MJ3h	CP_TD	60	49	65	21	16	5
AP_DARS	AP_T1	49	54	72	41	18	23
AP_DARS	ZRANK2	49	68	78	39	10	29
AP_DARS	PISA	49	55	71	38	16	22
AP_DARS	AP_T2	49	56	71	37	15	22
AP_DARS	PYDOCK_TOT	49	54	69	35	15	20
AP_DARS	FIREDOCK_AB	49	47	68	40	21	19
AP_DARS	FIREDOCK	49	50	66	33	16	17
AP_DARS	CP_SKOIP	49	56	64	23	8	15
AP_DARS	CP_BFKV	49	54	74	45	20	25
AP_DARS	FIREDOCK_EI	49	47	65	34	18	16
AP_DARS	CP_DECK	49	55	71	38	16	22
AP_DARS	AP_OPUS_PSP	49	60	74	39	14	25
AP_DARS	ZRANK	49	43	69	46	26	20
AP_DARS	CP_BT	49	46	69	43	23	20
AP_DARS	CP_TSC	49	58	77	47	19	28
AP_DARS	CP_RMFCA	49	40	62	35	22	13
AP_DARS	CP_TEl	49	50	70	41	20	21
AP_DARS	CP_TD	49	49	69	40	20	20
AP_DARS	CP_MJ3h	49	60	71	33	11	22
CP_SKOb	AP_T1	51	54	73	41	19	22
CP_SKOb	ZRANK2	51	68	77	35	9	26
CP_SKOb	PISA	51	55	70	34	15	19
CP_SKOb	AP_T2	51	56	71	35	15	20
CP_SKOb	PYDOCK_TOT	51	54	74	43	20	23
CP_SKOb	FIREDOCK_AB	51	47	72	46	25	21
CP_SKOb	FIREDOCK	51	50	73	45	23	22
CP_SKOb	CP_SKOIP	51	56	70	33	14	19
CP_SKOb	CP_BFKV	51	54	62	19	8	11

Table continued on next page

Table continued from previous page

Method A	Method B	$\|A\|$	$\|B\|$	$\|A \cup B\|$	$\|A \ominus B\|$	$\|A \setminus B\|$	$\|B \setminus A\|$
CP_SKOb	FIREDOCK_EI	51	47	73	48	26	22
CP_SKOb	CP_DECK	51	55	71	36	16	20
CP_SKOb	AP_OPUS_PSP	51	60	72	33	12	21
CP_SKOb	ZRANK	51	43	71	48	28	20
CP_SKOb	CP_BT	51	46	64	31	18	13
CP_SKOb	CP_TSC	51	58	73	37	15	22
CP_SKOb	CP_RMFCA	51	40	64	37	24	13
CP_SKOb	CP_TEl	51	50	67	33	17	16
CP_SKOb	CP_TD	51	49	66	32	17	15
CP_SKOb	CP_MJ3h	51	60	73	35	13	22
CP_SKOb	AP_DARS	51	49	68	36	19	17
CP_TB	AP_T1	50	54	66	28	12	16
CP_TB	ZRANK2	50	68	73	28	5	23
CP_TB	PISA	50	55	68	31	13	18
CP_TB	AP_T2	50	56	67	28	11	17
CP_TB	PYDOCK_TOT	50	54	66	28	12	16
CP_TB	FIREDOCK_AB	50	47	61	25	14	11
CP_TB	FIREDOCK	50	50	65	30	15	15
CP_TB	CP_SKOIP	50	56	70	34	14	20
CP_TB	CP_BFKV	50	54	68	32	14	18
CP_TB	FIREDOCK_EI	50	47	66	35	19	16
CP_TB	CP_DECK	50	55	71	37	16	21
CP_TB	AP_OPUS_PSP	50	60	69	28	9	19
CP_TB	ZRANK	50	43	60	27	17	10
CP_TB	CP_BT	50	46	62	28	16	12
CP_TB	CP_TSC	50	58	68	28	10	18
CP_TB	CP_RMFCA	50	40	59	28	19	9
CP_TB	CP_TEl	50	50	64	28	14	14
CP_TB	CP_TD	50	49	65	31	16	15
CP_TB	CP_MJ3h	50	60	67	24	7	17
CP_TB	AP_DARS	50	49	70	41	21	20
CP_TB	CP_SKOb	50	51	68	35	17	18
SPIDER	AP_T1	41	54	65	35	11	24
SPIDER	ZRANK2	41	68	77	45	9	36
SPIDER	PISA	41	55	65	34	10	24
SPIDER	AP_T2	41	56	65	33	9	24
SPIDER	PYDOCK_TOT	41	54	67	39	13	26
SPIDER	FIREDOCK_AB	41	47	59	30	12	18
SPIDER	FIREDOCK	41	50	62	33	12	21
SPIDER	CP_SKOIP	41	56	74	51	18	33
SPIDER	CP_BFKV	41	54	72	49	18	31
SPIDER	FIREDOCK_EI	41	47	61	34	14	20
SPIDER	CP_DECK	41	55	73	50	18	32
SPIDER	AP_OPUS_PSP	41	60	72	43	12	31
SPIDER	ZRANK	41	43	55	26	12	14
SPIDER	CP_BT	41	46	60	33	14	19
SPIDER	CP_TSC	41	58	69	39	11	28
SPIDER	CP_RMFCA	41	40	55	29	15	14
SPIDER	CP_TEl	41	50	69	47	19	28
SPIDER	CP_TD	41	49	64	38	15	23
SPIDER	CP_MJ3h	41	60	69	37	9	28
SPIDER	AP_DARS	41	49	67	44	18	26
SPIDER	CP_SKOb	41	51	74	56	23	33
SPIDER	CP_TB	41	50	66	41	16	25
SIPPER	AP_T1	56	54	73	36	19	17
SIPPER	ZRANK2	56	68	76	28	8	20
SIPPER	PISA	56	55	74	37	19	18
SIPPER	AP_T2	56	56	69	26	13	13
SIPPER	PYDOCK_TOT	56	54	69	28	15	13
SIPPER	FIREDOCK_AB	56	47	71	39	24	15
SIPPER	FIREDOCK	56	50	71	36	21	15
SIPPER	CP_SKOIP	56	56	73	34	17	17
SIPPER	CP_BFKV	56	54	69	28	15	13
SIPPER	FIREDOCK_EI	56	47	71	39	24	15
SIPPER	CP_DECK	56	55	74	37	19	18
SIPPER	AP_OPUS_PSP	56	60	75	34	15	19
SIPPER	ZRANK	56	43	69	39	26	13
SIPPER	CP_BT	56	46	67	32	21	11

Table continued on next page

Table continued from previous page

Method A	Method B	$\|A\|$	$\|B\|$	$\|A \cup B\|$	$\|A \ominus B\|$	$\|A \backslash B\|$	$\|B \backslash A\|$
SIPPER	CP_TSC	56	58	75	36	17	19
SIPPER	CP_RMFCA	56	40	64	32	24	8
SIPPER	CP_TEl	56	50	71	36	21	15
SIPPER	CP_TD	56	49	69	33	20	13
SIPPER	CP_MJ3h	56	60	73	30	13	17
SIPPER	AP_DARS	56	49	73	41	24	17
SIPPER	CP_SKOb	56	51	68	29	17	12
SIPPER	CP_TB	56	50	67	28	17	11
SIPPER	SPIDER	56	41	71	45	30	15
AP_DCOMPLEX	AP_T1	37	54	57	23	3	20
AP_DCOMPLEX	ZRANK2	37	68	69	33	1	32
AP_DCOMPLEX	PISA	37	55	59	26	4	22
AP_DCOMPLEX	AP_T2	37	56	61	29	5	24
AP_DCOMPLEX	PYDOCK_TOT	37	54	64	37	10	27
AP_DCOMPLEX	FIREDOCK_AB	37	47	53	22	6	16
AP_DCOMPLEX	FIREDOCK	37	50	57	27	7	20
AP_DCOMPLEX	CP_SKOIP	37	56	67	41	11	30
AP_DCOMPLEX	CP_BFKV	37	54	70	49	16	33
AP_DCOMPLEX	FIREDOCK_EI	37	47	56	28	9	19
AP_DCOMPLEX	CP_DECK	37	55	67	42	12	30
AP_DCOMPLEX	AP_OPUS_PSP	37	60	64	31	4	27
AP_DCOMPLEX	ZRANK	37	43	48	16	5	11
AP_DCOMPLEX	CP_BT	37	46	58	33	12	21
AP_DCOMPLEX	CP_TSC	37	58	61	27	3	24
AP_DCOMPLEX	CP_RMFCA	37	40	49	21	9	12
AP_DCOMPLEX	CP_TEl	37	50	62	37	12	25
AP_DCOMPLEX	CP_TD	37	49	61	36	12	24
AP_DCOMPLEX	CP_MJ3h	37	60	68	39	8	31
AP_DCOMPLEX	AP_DARS	37	49	65	44	16	28
AP_DCOMPLEX	CP_SKOb	37	51	67	46	16	30
AP_DCOMPLEX	CP_TB	37	50	59	31	9	22
AP_DCOMPLEX	SPIDER	37	41	53	28	12	16
AP_DCOMPLEX	SIPPER	37	56	69	45	13	32
CP_Qp	AP_T1	49	54	68	33	14	19
CP_Qp	ZRANK2	49	68	72	27	4	23
CP_Qp	PISA	49	55	66	28	11	17
CP_Qp	AP_T2	49	56	69	33	13	20
CP_Qp	PYDOCK_TOT	49	54	70	37	16	21
CP_Qp	FIREDOCK_AB	49	47	63	30	16	14
CP_Qp	FIREDOCK	49	50	67	35	17	18
CP_Qp	CP_SKOIP	49	56	68	31	12	19
CP_Qp	CP_BFKV	49	54	69	35	15	20
CP_Qp	FIREDOCK_EI	49	47	65	34	18	16
CP_Qp	CP_DECK	49	55	69	34	14	20
CP_Qp	AP_OPUS_PSP	49	60	65	21	5	16
CP_Qp	ZRANK	49	43	61	30	18	12
CP_Qp	CP_BT	49	46	64	33	18	15
CP_Qp	CP_TSC	49	58	67	27	9	18
CP_Qp	CP_RMFCA	49	40	59	29	19	10
CP_Qp	CP_TEl	49	50	64	29	14	15
CP_Qp	CP_TD	49	49	63	28	14	14
CP_Qp	CP_MJ3h	49	60	69	29	9	20
CP_Qp	AP_DARS	49	49	70	42	21	21
CP_Qp	CP_SKOb	49	51	63	26	12	14
CP_Qp	CP_TB	49	50	62	25	12	13
CP_Qp	SPIDER	49	41	67	44	26	18
CP_Qp	SIPPER	49	56	69	33	13	20
CP_Qp	AP_DCOMPLEX	49	37	57	28	20	8
CP_HLPL	AP_T1	52	54	68	30	14	16
CP_HLPL	ZRANK2	52	68	71	22	3	19
CP_HLPL	PISA	52	55	66	25	11	14
CP_HLPL	AP_T2	52	56	68	28	12	16
CP_HLPL	PYDOCK_TOT	52	54	69	32	15	17
CP_HLPL	FIREDOCK_AB	52	47	62	25	15	10
CP_HLPL	FIREDOCK	52	50	66	30	16	14
CP_HLPL	CP_SKOIP	52	56	68	28	12	16
CP_HLPL	CP_BFKV	52	54	70	34	16	18
CP_HLPL	FIREDOCK_EI	52	47	65	31	18	13

Table continued on next page

Table continued from previous page

Method A	Method B	$\|A\|$	$\|B\|$	$\|A \cup B\|$	$\|A \ominus B\|$	$\|A \backslash B\|$	$\|B \backslash A\|$
CP_HLPL	CP_DECK	52	55	69	31	14	17
CP_HLPL	AP_OPUS_PSP	52	60	64	16	4	12
CP_HLPL	ZRANK	52	43	62	29	19	10
CP_HLPL	CP_BT	52	46	63	28	17	11
CP_HLPL	CP_TSC	52	58	68	26	10	16
CP_HLPL	CP_RMFCA	52	40	58	24	18	6
CP_HLPL	CP_TEl	52	50	66	30	16	14
CP_HLPL	CP_TD	52	49	63	25	14	11
CP_HLPL	CP_MJ3h	52	60	68	24	8	16
CP_HLPL	AP_DARS	52	49	70	39	21	18
CP_HLPL	CP_SKOb	52	51	66	29	15	14
CP_HLPL	CP_TB	52	50	60	18	10	8
CP_HLPL	SPIDER	52	41	67	41	26	15
CP_HLPL	SIPPER	52	56	67	26	11	15
CP_HLPL	AP_DCOMPLEX	52	37	59	29	22	7
CP_HLPL	CP_Qp	52	49	55	9	6	3
CHARMM_ELE	AP_T1	39	54	67	41	13	28
CHARMM_ELE	ZRANK2	39	68	76	45	8	37
CHARMM_ELE	PISA	39	55	67	40	12	28
CHARMM_ELE	AP_T2	39	56	66	37	10	27
CHARMM_ELE	PYDOCK_TOT	39	54	70	47	16	31
CHARMM_ELE	FIREDOCK_AB	39	47	61	36	14	22
CHARMM_ELE	FIREDOCK	39	50	66	43	16	27
CHARMM_ELE	CP_SKOIP	39	56	76	57	20	37
CHARMM_ELE	CP_BFKV	39	54	68	43	14	29
CHARMM_ELE	FIREDOCK_EI	39	47	66	46	19	27
CHARMM_ELE	CP_DECK	39	55	72	50	17	33
CHARMM_ELE	AP_OPUS_PSP	39	60	74	49	14	35
CHARMM_ELE	ZRANK	39	43	57	32	14	18
CHARMM_ELE	CP_BT	39	46	62	39	16	23
CHARMM_ELE	CP_TSC	39	58	71	45	13	32
CHARMM_ELE	CP_RMFCA	39	40	60	41	20	21
CHARMM_ELE	CP_TEl	39	50	67	45	17	28
CHARMM_ELE	CP_TD	39	49	68	48	19	29
CHARMM_ELE	CP_MJ3h	39	60	76	53	16	37
CHARMM_ELE	AP_DARS	39	49	73	58	24	34
CHARMM_ELE	CP_SKOb	39	51	65	40	14	26
CHARMM_ELE	CP_TB	39	50	66	43	16	27
CHARMM_ELE	SPIDER	39	41	62	44	21	23
CHARMM_ELE	SIPPER	39	56	72	49	16	33
CHARMM_ELE	AP_DCOMPLEX	39	37	58	40	21	19
CHARMM_ELE	CP_Qp	39	49	66	44	17	27
CHARMM_ELE	CP_HLPL	39	52	69	47	17	30
CP_MIXRANK_MIN	AP_T1	42	54	67	38	13	25
CP_MIXRANK_MIN	ZRANK2	42	68	76	42	8	34
CP_MIXRANK_MIN	PISA	42	55	68	39	13	26
CP_MIXRANK_MIN	AP_T2	42	56	69	40	13	27
CP_MIXRANK_MIN	PYDOCK_TOT	42	54	72	48	18	30
CP_MIXRANK_MIN	FIREDOCK_AB	42	47	66	43	19	24
CP_MIXRANK_MIN	FIREDOCK	42	50	67	42	17	25
CP_MIXRANK_MIN	CP_SKOIP	42	56	68	38	12	26
CP_MIXRANK_MIN	CP_BFKV	42	54	66	36	12	24
CP_MIXRANK_MIN	FIREDOCK_EI	42	47	66	43	19	24
CP_MIXRANK_MIN	CP_DECK	42	55	68	39	13	26
CP_MIXRANK_MIN	AP_OPUS_PSP	42	60	73	44	13	31
CP_MIXRANK_MIN	ZRANK	42	43	64	43	21	22
CP_MIXRANK_MIN	CP_BT	42	46	62	36	16	20
CP_MIXRANK_MIN	CP_TSC	42	58	72	44	14	30
CP_MIXRANK_MIN	CP_RMFCA	42	40	61	40	21	19
CP_MIXRANK_MIN	CP_TEl	42	50	64	36	14	22
CP_MIXRANK_MIN	CP_TD	42	49	61	31	12	19
CP_MIXRANK_MIN	CP_MJ3h	42	60	68	34	8	26
CP_MIXRANK_MIN	AP_DARS	42	49	69	47	20	27
CP_MIXRANK_MIN	CP_SKOb	42	51	64	35	13	22
CP_MIXRANK_MIN	CP_TB	42	50	65	38	15	23
CP_MIXRANK_MIN	SPIDER	42	41	63	43	22	21
CP_MIXRANK_MIN	SIPPER	42	56	67	36	11	25
CP_MIXRANK_MIN	AP_DCOMPLEX	42	37	59	39	22	17

Table continued on next page

Table continued from previous page

Method A	Method B	$\|A\|$	$\|B\|$	$\|A \cup B\|$	$\|A \ominus B\|$	$\|A \backslash B\|$	$\|B \backslash A\|$
CP_MIXRANK_MIN	CP_Qp	42	49	61	31	12	19
CP_MIXRANK_MIN	CP_HLPL	42	52	63	32	11	21
CP_MIXRANK_MIN	CHARMM_ELE	42	39	63	45	24	21
CP_ES3DC_MIN	AP_T1	50	54	73	42	19	23
CP_ES3DC_MIN	ZRANK2	50	68	78	38	10	28
CP_ES3DC_MIN	PISA	50	55	73	41	18	23
CP_ES3DC_MIN	AP_T2	50	56	74	42	18	24
CP_ES3DC_MIN	PYDOCK_TOT	50	54	72	40	18	22
CP_ES3DC_MIN	FIREDOCK_AB	50	47	71	45	24	21
CP_ES3DC_MIN	FIREDOCK	50	50	74	48	24	24
CP_ES3DC_MIN	CP_SKOIP	50	56	72	38	16	22
CP_ES3DC_MIN	CP_BFKV	50	54	69	34	15	19
CP_ES3DC_MIN	FIREDOCK_EI	50	47	74	51	27	24
CP_ES3DC_MIN	CP_DECK	50	55	72	39	17	22
CP_ES3DC_MIN	AP_OPUS_PSP	50	60	74	38	14	24
CP_ES3DC_MIN	ZRANK	50	43	69	45	26	19
CP_ES3DC_MIN	CP_BT	50	46	66	36	20	16
CP_ES3DC_MIN	CP_TSC	50	58	74	40	16	24
CP_ES3DC_MIN	CP_RMFCA	50	40	66	42	26	16
CP_ES3DC_MIN	CP_TEl	50	50	71	42	21	21
CP_ES3DC_MIN	CP_TD	50	49	70	41	21	20
CP_ES3DC_MIN	CP_MJ3h	50	60	75	40	15	25
CP_ES3DC_MIN	AP_DARS	50	49	74	49	25	24
CP_ES3DC_MIN	CP_SKOb	50	51	65	29	14	15
CP_ES3DC_MIN	CP_TB	50	50	67	34	17	17
CP_ES3DC_MIN	SPIDER	50	41	74	57	33	24
CP_ES3DC_MIN	SIPPER	50	56	67	28	11	17
CP_ES3DC_MIN	AP_DCOMPLEX	50	37	67	47	30	17
CP_ES3DC_MIN	CP_Qp	50	49	65	31	16	15
CP_ES3DC_MIN	CP_HLPL	50	52	66	30	14	16
CP_ES3DC_MIN	CHARMM_ELE	50	39	66	43	27	16
CP_ES3DC_MIN	CP_MIXRANK_MIN	50	42	58	24	16	8
CP_ZS3DC_MIN	AP_T1	48	54	70	38	16	22
CP_ZS3DC_MIN	ZRANK2	48	68	76	36	8	28
CP_ZS3DC_MIN	PISA	48	55	72	41	17	24
CP_ZS3DC_MIN	AP_T2	48	56	72	40	16	24
CP_ZS3DC_MIN	PYDOCK_TOT	48	54	69	36	15	21
CP_ZS3DC_MIN	FIREDOCK_AB	48	47	68	41	21	20
CP_ZS3DC_MIN	FIREDOCK	48	50	71	44	21	23
CP_ZS3DC_MIN	CP_SKOIP	48	56	71	38	15	23
CP_ZS3DC_MIN	CP_BFKV	48	54	67	32	13	19
CP_ZS3DC_MIN	FIREDOCK_EI	48	47	71	47	24	23
CP_ZS3DC_MIN	CP_DECK	48	55	72	41	17	24
CP_ZS3DC_MIN	AP_OPUS_PSP	48	60	73	38	13	25
CP_ZS3DC_MIN	ZRANK	48	43	66	41	23	18
CP_ZS3DC_MIN	CP_BT	48	46	63	32	17	15
CP_ZS3DC_MIN	CP_TSC	48	58	72	38	14	24
CP_ZS3DC_MIN	CP_RMFCA	48	40	63	38	23	15
CP_ZS3DC_MIN	CP_TEl	48	50	68	38	18	20
CP_ZS3DC_MIN	CP_TD	48	49	68	39	19	20
CP_ZS3DC_MIN	CP_MJ3h	48	60	72	36	12	24
CP_ZS3DC_MIN	AP_DARS	48	49	72	47	23	24
CP_ZS3DC_MIN	CP_SKOb	48	51	64	29	13	16
CP_ZS3DC_MIN	CP_TB	48	50	64	30	14	16
CP_ZS3DC_MIN	SPIDER	48	41	71	53	30	23
CP_ZS3DC_MIN	SIPPER	48	56	66	28	10	18
CP_ZS3DC_MIN	AP_DCOMPLEX	48	37	64	43	27	16
CP_ZS3DC_MIN	CP_Qp	48	49	63	29	14	15
CP_ZS3DC_MIN	CP_HLPL	48	52	64	28	12	16
CP_ZS3DC_MIN	CHARMM_ELE	48	39	63	39	24	15
CP_ZS3DC_MIN	CP_MIXRANK_MIN	48	42	56	22	14	8
CP_ZS3DC_MIN	CP_ES3DC_MIN	48	50	51	4	1	3
AP_dDFIRE	AP_T1	33	54	56	25	2	23
AP_dDFIRE	ZRANK2	33	68	68	35	0	35
AP_dDFIRE	PISA	33	55	58	28	3	25
AP_dDFIRE	AP_T2	33	56	59	29	3	26
AP_dDFIRE	PYDOCK_TOT	33	54	61	35	7	28
AP_dDFIRE	FIREDOCK_AB	33	47	52	24	5	19

Table continued on next page

Table continued from previous page

Method A	Method B	$\|A\|$	$\|B\|$	$\|A \cup B\|$	$\|A \ominus B\|$	$\|A \backslash B\|$	$\|B \backslash A\|$
AP_dDFIRE	FIREDOCK	33	50	55	27	5	22
AP_dDFIRE	CP_SKOIP	33	56	64	39	8	31
AP_dDFIRE	CP_BFKV	33	54	66	45	12	33
AP_dDFIRE	FIREDOCK_EI	33	47	53	26	6	20
AP_dDFIRE	CP_DECK	33	55	64	40	9	31
AP_dDFIRE	AP_OPUS_PSP	33	60	61	29	1	28
AP_dDFIRE	ZRANK	33	43	49	22	6	16
AP_dDFIRE	CP_BT	33	46	54	29	8	21
AP_dDFIRE	CP_TSC	33	58	62	33	4	29
AP_dDFIRE	CP_RMFCA	33	40	44	15	4	11
AP_dDFIRE	CP_TEl	33	50	61	39	11	28
AP_dDFIRE	CP_TD	33	49	57	32	8	24
AP_dDFIRE	CP_MJ3h	33	60	65	37	5	32
AP_dDFIRE	AP_DARS	33	49	59	36	10	26
AP_dDFIRE	CP_SKOb	33	51	63	42	12	30
AP_dDFIRE	CP_TB	33	50	59	35	9	26
AP_dDFIRE	SPIDER	33	41	50	26	9	17
AP_dDFIRE	SIPPER	33	56	63	37	7	30
AP_dDFIRE	AP_DCOMPLEX	33	37	42	14	5	9
AP_dDFIRE	CP_Qp	33	49	55	28	6	22
AP_dDFIRE	CP_HLPL	33	52	55	25	3	22
AP_dDFIRE	CHARMM_ELE	33	39	56	40	17	23
AP_dDFIRE	CP_MIXRANK_MIN	33	42	56	37	14	23
AP_dDFIRE	CP_ES3DC_MIN	33	50	65	47	15	32
AP_dDFIRE	CP_ZS3DC_MIN	33	48	62	43	14	29
CP_SKOa	AP_T1	52	54	74	42	20	22
CP_SKOa	ZRANK2	52	68	79	38	11	27
CP_SKOa	PISA	52	55	71	35	16	19
CP_SKOa	AP_T2	52	56	73	38	17	21
CP_SKOa	PYDOCK_TOT	52	54	74	42	20	22
CP_SKOa	FIREDOCK_AB	52	47	74	49	27	22
CP_SKOa	FIREDOCK	52	50	76	50	26	24
CP_SKOa	CP_SKOIP	52	56	72	36	16	20
CP_SKOa	CP_BFKV	52	54	60	14	6	8
CP_SKOa	FIREDOCK_EI	52	47	74	49	27	22
CP_SKOa	CP_DECK	52	55	74	41	19	22
CP_SKOa	AP_OPUS_PSP	52	60	74	36	14	22
CP_SKOa	ZRANK	52	43	73	51	30	21
CP_SKOa	CP_BT	52	46	64	30	18	12
CP_SKOa	CP_TSC	52	58	76	42	18	24
CP_SKOa	CP_RMFCA	52	40	66	40	26	14
CP_SKOa	CP_TEl	52	50	70	38	20	18
CP_SKOa	CP_TD	52	49	69	37	20	17
CP_SKOa	CP_MJ3h	52	60	74	36	14	22
CP_SKOa	AP_DARS	52	49	70	39	21	18
CP_SKOa	CP_SKOb	52	51	58	13	7	6
CP_SKOa	CP_TB	52	50	69	36	19	17
CP_SKOa	SPIDER	52	41	76	59	35	24
CP_SKOa	SIPPER	52	56	69	30	13	17
CP_SKOa	AP_DCOMPLEX	52	37	70	51	33	18
CP_SKOa	CP_Qp	52	49	66	31	17	14
CP_SKOa	CP_HLPL	52	52	68	32	16	16
CP_SKOa	CHARMM_ELE	52	39	69	47	30	17
CP_SKOa	CP_MIXRANK_MIN	52	42	63	32	21	11
CP_SKOa	CP_ES3DC_MIN	52	50	64	26	14	12
CP_SKOa	CP_ZS3DC_MIN	52	48	64	28	16	12
CP_SKOa	AP_dDFIRE	52	33	67	49	34	15
CHARMM_TOT	AP_T1	49	54	69	35	15	20
CHARMM_TOT	ZRANK2	49	68	80	43	12	31
CHARMM_TOT	PISA	49	55	74	44	19	25
CHARMM_TOT	AP_T2	49	56	70	35	14	21
CHARMM_TOT	PYDOCK_TOT	49	54	66	29	12	17
CHARMM_TOT	FIREDOCK_AB	49	47	67	38	20	18
CHARMM_TOT	FIREDOCK	49	50	66	33	16	17
CHARMM_TOT	CP_SKOIP	49	56	71	37	15	22
CHARMM_TOT	CP_BFKV	49	54	75	47	21	26
CHARMM_TOT	FIREDOCK_EI	49	47	66	36	19	17
CHARMM_TOT	CP_DECK	49	55	70	36	15	21

Table continued on next page

Table continued from previous page

Method A	Method B	$\|A\|$	$\|B\|$	$\|A \cup B\|$	$\|A \ominus B\|$	$\|A \backslash B\|$	$\|B \backslash A\|$
CHARMM_TOT	AP_OPUS_PSP	49	60	75	41	15	26
CHARMM_TOT	ZRANK	49	43	66	40	23	17
CHARMM_TOT	CP_BT	49	46	67	39	21	18
CHARMM_TOT	CP_TSC	49	58	75	43	17	26
CHARMM_TOT	CP_RMFCA	49	40	63	37	23	14
CHARMM_TOT	CP_TEl	49	50	70	41	20	21
CHARMM_TOT	CP_TD	49	49	69	40	20	20
CHARMM_TOT	CP_MJ3h	49	60	71	33	11	22
CHARMM_TOT	AP_DARS	49	49	67	36	18	18
CHARMM_TOT	CP_SKOb	49	51	71	42	20	22
CHARMM_TOT	CP_TB	49	50	69	39	19	20
CHARMM_TOT	SPIDER	49	41	69	48	28	20
CHARMM_TOT	SIPPER	49	56	75	45	19	26
CHARMM_TOT	AP_DCOMPLEX	49	37	63	40	26	14
CHARMM_TOT	CP_Qp	49	49	71	44	22	22
CHARMM_TOT	CP_HLPL	49	52	73	45	21	24
CHARMM_TOT	CHARMM_ELE	49	39	68	48	29	19
CHARMM_TOT	CP_MIXRANK_MIN	49	42	68	45	26	19
CHARMM_TOT	CP_ES3DC_MIN	49	50	72	45	22	23
CHARMM_TOT	CP_ZS3DC_MIN	49	48	71	45	23	22
CHARMM_TOT	AP_dDFIRE	49	33	62	42	29	13
CHARMM_TOT	CP_SKOa	49	52	73	45	21	24
ROSETTADOCK	AP_T1	41	54	59	23	5	18
ROSETTADOCK	ZRANK2	41	68	70	31	2	29
ROSETTADOCK	PISA	41	55	63	30	8	22
ROSETTADOCK	AP_T2	41	56	63	29	7	22
ROSETTADOCK	PYDOCK_TOT	41	54	64	33	10	23
ROSETTADOCK	FIREDOCK_AB	41	47	56	24	9	15
ROSETTADOCK	FIREDOCK	41	50	59	27	9	18
ROSETTADOCK	CP_SKOIP	41	56	67	37	11	26
ROSETTADOCK	CP_BFKV	41	54	70	45	16	29
ROSETTADOCK	FIREDOCK_EI	41	47	56	24	9	15
ROSETTADOCK	CP_DECK	41	55	70	44	15	29
ROSETTADOCK	AP_OPUS_PSP	41	60	65	29	5	24
ROSETTADOCK	ZRANK	41	43	52	20	9	11
ROSETTADOCK	CP_BT	41	46	62	37	16	21
ROSETTADOCK	CP_TSC	41	58	65	31	7	24
ROSETTADOCK	CP_RMFCA	41	40	49	17	9	8
ROSETTADOCK	CP_TEl	41	50	64	37	14	23
ROSETTADOCK	CP_TD	41	49	66	42	17	25
ROSETTADOCK	CP_MJ3h	41	60	70	39	10	29
ROSETTADOCK	AP_DARS	41	49	64	38	15	23
ROSETTADOCK	CP_SKOb	41	51	68	44	17	27
ROSETTADOCK	CP_TB	41	50	61	31	11	20
ROSETTADOCK	SPIDER	41	41	57	32	16	16
ROSETTADOCK	SIPPER	41	56	69	41	13	28
ROSETTADOCK	AP_DCOMPLEX	41	37	48	18	11	7
ROSETTADOCK	CP_Qp	41	49	61	32	12	20
ROSETTADOCK	CP_HLPL	41	52	62	31	10	21
ROSETTADOCK	CHARMM_ELE	41	39	62	44	23	21
ROSETTADOCK	CP_MIXRANK_MIN	41	42	62	41	20	21
ROSETTADOCK	CP_ES3DC_MIN	41	50	68	45	18	27
ROSETTADOCK	CP_ZS3DC_MIN	41	48	65	41	17	24
ROSETTADOCK	AP_dDFIRE	41	33	46	18	13	5
ROSETTADOCK	CP_SKOa	41	52	70	47	18	29
ROSETTADOCK	CHARMM_TOT	41	49	65	40	16	24
ATTRACT	AP_T1	38	54	57	22	3	19
ATTRACT	ZRANK2	38	68	69	32	1	31
ATTRACT	PISA	38	55	59	25	4	21
ATTRACT	AP_T2	38	56	60	26	4	22
ATTRACT	PYDOCK_TOT	38	54	64	36	10	26
ATTRACT	FIREDOCK_AB	38	47	53	21	6	15
ATTRACT	FIREDOCK	38	50	58	28	8	20
ATTRACT	CP_SKOIP	38	56	68	42	12	30
ATTRACT	CP_BFKV	38	54	68	44	14	30
ATTRACT	FIREDOCK_EI	38	47	56	27	9	18
ATTRACT	CP_DECK	38	55	68	43	13	30
ATTRACT	AP_OPUS_PSP	38	60	64	30	4	26

Table continued on next page

Table continued from previous page

Method A	Method B	$\lvert A\rvert$	$\lvert B\rvert$	$\lvert A\cup B\rvert$	$\lvert A\ominus B\rvert$	$\lvert A\backslash B\rvert$	$\lvert B\backslash A\rvert$
ATTRACT	ZRANK	38	43	50	19	7	12
ATTRACT	CP_BT	38	46	58	32	12	20
ATTRACT	CP_TSC	38	58	63	30	5	25
ATTRACT	CP_RMFCA	38	40	49	20	9	11
ATTRACT	CP_TEl	38	50	61	34	11	23
ATTRACT	CP_TD	38	49	61	35	12	23
ATTRACT	CP_MJ3h	38	60	68	38	8	30
ATTRACT	AP_DARS	38	49	66	45	17	28
ATTRACT	CP_SKOb	38	51	66	43	15	28
ATTRACT	CP_TB	38	50	59	30	9	21
ATTRACT	SPIDER	38	41	55	31	14	17
ATTRACT	SIPPER	38	56	67	40	11	29
ATTRACT	AP_DCOMPLEX	38	37	42	9	5	4
ATTRACT	CP_Qp	38	49	57	27	8	19
ATTRACT	CP_HLPL	38	52	59	28	7	21
ATTRACT	CHARMM_ELE	38	39	57	37	18	19
ATTRACT	CP_MIXRANK_MIN	38	42	57	34	15	19
ATTRACT	CP_ES3DC_MIN	38	50	65	42	15	27
ATTRACT	CP_ZS3DC_MIN	38	48	62	38	14	24
ATTRACT	AP_dDFIRE	38	33	43	15	10	5
ATTRACT	CP_SKOa	38	52	69	48	17	31
ATTRACT	CHARMM_TOT	38	49	64	41	15	26
ATTRACT	ROSETTADOCK	38	41	49	19	8	11
PROPNSTS	AP_T1	51	54	74	43	20	23
PROPNSTS	ZRANK2	51	68	76	33	8	25
PROPNSTS	PISA	51	55	73	40	18	22
PROPNSTS	AP_T2	51	56	74	41	18	23
PROPNSTS	PYDOCK_TOT	51	54	71	37	17	20
PROPNSTS	FIREDOCK_AB	51	47	73	48	26	22
PROPNSTS	FIREDOCK	51	50	74	47	24	23
PROPNSTS	CP_SKOIP	51	56	71	35	15	20
PROPNSTS	CP_BFKV	51	54	67	29	13	16
PROPNSTS	FIREDOCK_EI	51	47	73	48	26	22
PROPNSTS	CP_DECK	51	55	73	40	18	22
PROPNSTS	AP_OPUS_PSP	51	60	74	37	14	23
PROPNSTS	ZRANK	51	43	72	50	29	21
PROPNSTS	CP_BT	51	46	67	37	21	16
PROPNSTS	CP_TSC	51	58	75	41	17	24
PROPNSTS	CP_RMFCA	51	40	66	41	26	15
PROPNSTS	CP_TEl	51	50	68	35	18	17
PROPNSTS	CP_TD	51	49	70	40	21	19
PROPNSTS	CP_MJ3h	51	60	72	33	12	21
PROPNSTS	AP_DARS	51	49	72	44	23	21
PROPNSTS	CP_SKOb	51	51	63	24	12	12
PROPNSTS	CP_TB	51	50	67	33	17	16
PROPNSTS	SPIDER	51	41	77	62	36	26
PROPNSTS	SIPPER	51	56	66	25	10	15
PROPNSTS	AP_DCOMPLEX	51	37	71	54	34	20
PROPNSTS	CP_Qp	51	49	65	30	16	14
PROPNSTS	CP_HLPL	51	52	66	29	14	15
PROPNSTS	CHARMM_ELE	51	39	68	46	29	17
PROPNSTS	CP_MIXRANK_MIN	51	42	66	39	24	15
PROPNSTS	CP_ES3DC_MIN	51	50	65	29	15	14
PROPNSTS	CP_ZS3DC_MIN	51	48	64	29	16	13
PROPNSTS	AP_dDFIRE	51	33	67	50	34	16
PROPNSTS	CP_SKOa	51	52	63	23	11	12
PROPNSTS	CHARMM_TOT	51	49	74	48	25	23
PROPNSTS	ROSETTADOCK	51	41	72	52	31	21
PROPNSTS	ATTRACT	51	38	69	49	31	18
DESOLV	AP_T1	46	54	70	40	16	24
DESOLV	ZRANK2	46	68	74	34	6	28
DESOLV	PISA	46	55	71	41	16	25
DESOLV	AP_T2	46	56	70	38	14	24
DESOLV	PYDOCK_TOT	46	54	63	26	9	17
DESOLV	FIREDOCK_AB	46	47	68	43	21	22
DESOLV	FIREDOCK	46	50	67	38	17	21
DESOLV	CP_SKOIP	46	56	66	30	10	20
DESOLV	CP_BFKV	46	54	72	44	18	26

Table continued on next page

Table continued from previous page

Method A	Method B	$\|A\|$	$\|B\|$	$\|A \cup B\|$	$\|A \ominus B\|$	$\|A \backslash B\|$	$\|B \backslash A\|$
DESOLV	FIREDOCK_EI	46	47	66	39	19	20
DESOLV	CP_DECK	46	55	67	33	12	21
DESOLV	AP_OPUS_PSP	46	60	69	32	9	23
DESOLV	ZRANK	46	43	67	45	24	21
DESOLV	CP_BT	46	46	66	40	20	20
DESOLV	CP_TSC	46	58	71	38	13	25
DESOLV	CP_RMFCA	46	40	61	36	21	15
DESOLV	CP_TEl	46	50	67	38	17	21
DESOLV	CP_TD	46	49	66	37	17	20
DESOLV	CP_MJ3h	46	60	69	32	9	23
DESOLV	AP_DARS	46	49	59	23	10	13
DESOLV	CP_SKOb	46	51	66	35	15	20
DESOLV	CP_TB	46	50	66	36	16	20
DESOLV	SPIDER	46	41	67	47	26	21
DESOLV	SIPPER	46	56	68	34	12	22
DESOLV	AP_DCOMPLEX	46	37	62	41	25	16
DESOLV	CP_Qp	46	49	66	37	17	20
DESOLV	CP_HLPL	46	52	67	36	15	21
DESOLV	CHARMM_ELE	46	39	69	53	30	23
DESOLV	CP_MIXRANK_MIN	46	42	66	44	24	20
DESOLV	CP_ES3DC_MIN	46	50	66	36	16	20
DESOLV	CP_ZS3DC_MIN	46	48	65	36	17	19
DESOLV	AP_dDFIRE	46	33	59	39	26	13
DESOLV	CP_SKOa	46	52	66	34	14	20
DESOLV	CHARMM_TOT	46	49	60	25	11	14
DESOLV	ROSETTADOCK	46	41	63	39	22	17
DESOLV	ATTRACT	46	38	64	44	26	18
DESOLV	PROPNSTS	46	51	68	39	17	22
CP_Qm	AP_T1	49	54	73	43	19	24
CP_Qm	ZRANK2	49	68	78	39	10	29
CP_Qm	PISA	49	55	73	42	18	24
CP_Qm	AP_T2	49	56	74	43	18	25
CP_Qm	PYDOCK_TOT	49	54	71	39	17	22
CP_Qm	FIREDOCK_AB	49	47	72	48	25	23
CP_Qm	FIREDOCK	49	50	74	49	24	25
CP_Qm	CP_SKOIP	49	56	73	41	17	24
CP_Qm	CP_BFKV	49	54	64	25	10	15
CP_Qm	FIREDOCK_EI	49	47	71	46	24	22
CP_Qm	CP_DECK	49	55	72	40	17	23
CP_Qm	AP_OPUS_PSP	49	60	73	37	13	24
CP_Qm	ZRANK	49	43	69	46	26	20
CP_Qm	CP_BT	49	46	64	33	18	15
CP_Qm	CP_TSC	49	58	76	45	18	27
CP_Qm	CP_RMFCA	49	40	66	43	26	17
CP_Qm	CP_TEl	49	50	67	35	17	18
CP_Qm	CP_TD	49	49	66	34	17	17
CP_Qm	CP_MJ3h	49	60	70	31	10	21
CP_Qm	AP_DARS	49	49	70	42	21	21
CP_Qm	CP_SKOb	49	51	64	28	13	15
CP_Qm	CP_TB	49	50	66	33	16	17
CP_Qm	SPIDER	49	41	74	58	33	25
CP_Qm	SIPPER	49	56	70	35	14	21
CP_Qm	AP_DCOMPLEX	49	37	68	50	31	19
CP_Qm	CP_Qp	49	49	65	32	16	16
CP_Qm	CP_HLPL	49	52	67	33	15	18
CP_Qm	CHARMM_ELE	49	39	70	52	31	21
CP_Qm	CP_MIXRANK_MIN	49	42	63	35	21	14
CP_Qm	CP_ES3DC_MIN	49	50	65	31	15	16
CP_Qm	CP_ZS3DC_MIN	49	48	64	31	16	15
CP_Qm	AP_dDFIRE	49	33	67	52	34	18
CP_Qm	CP_SKOa	49	52	59	17	7	10
CP_Qm	CHARMM_TOT	49	49	69	40	20	20
CP_Qm	ROSETTADOCK	49	41	71	52	30	22
CP_Qm	ATTRACT	49	38	66	45	28	17
CP_Qm	PROPNSTS	49	51	63	26	12	14
CP_Qm	DESOLV	49	46	63	31	17	14
AP_GEOMETRIC	AP_T1	37	54	59	27	5	22
AP_GEOMETRIC	ZRANK2	37	68	69	33	1	32

Table continued on next page

Table continued from previous page

Method A	Method B	$\|A\|$	$\|B\|$	$\|A \cup B\|$	$\|A \ominus B\|$	$\|A \backslash B\|$	$\|B \backslash A\|$
AP_GEOMETRIC	PISA	37	55	62	32	7	25
AP_GEOMETRIC	AP_T2	37	56	62	31	6	25
AP_GEOMETRIC	PYDOCK_TOT	37	54	66	41	12	29
AP_GEOMETRIC	FIREDOCK_AB	37	47	57	30	10	20
AP_GEOMETRIC	FIREDOCK	37	50	61	35	11	24
AP_GEOMETRIC	CP_SKOIP	37	56	67	41	11	30
AP_GEOMETRIC	CP_BFKV	37	54	68	45	14	31
AP_GEOMETRIC	FIREDOCK_EI	37	47	60	36	13	23
AP_GEOMETRIC	CP_DECK	37	55	63	34	8	26
AP_GEOMETRIC	AP_OPUS_PSP	37	60	63	29	3	26
AP_GEOMETRIC	ZRANK	37	43	52	24	9	15
AP_GEOMETRIC	CP_BT	37	46	57	31	11	20
AP_GEOMETRIC	CP_TSC	37	58	62	29	4	25
AP_GEOMETRIC	CP_RMFCA	37	40	50	23	10	13
AP_GEOMETRIC	CP_TEl	37	50	63	39	13	26
AP_GEOMETRIC	CP_TD	37	49	60	34	11	23
AP_GEOMETRIC	CP_MJ3h	37	60	68	39	8	31
AP_GEOMETRIC	AP_DARS	37	49	67	48	18	30
AP_GEOMETRIC	CP_SKOb	37	51	66	44	15	29
AP_GEOMETRIC	CP_TB	37	50	59	31	9	22
AP_GEOMETRIC	SPIDER	37	41	55	32	14	18
AP_GEOMETRIC	SIPPER	37	56	67	41	11	30
AP_GEOMETRIC	AP_DCOMPLEX	37	37	45	16	8	8
AP_GEOMETRIC	CP_Qp	37	49	57	28	8	20
AP_GEOMETRIC	CP_HLPL	37	52	58	27	6	21
AP_GEOMETRIC	CHARMM_ELE	37	39	60	44	21	23
AP_GEOMETRIC	CP_MIXRANK_MIN	37	42	58	37	16	21
AP_GEOMETRIC	CP_ES3DC_MIN	37	50	65	43	15	28
AP_GEOMETRIC	CP_ZS3DC_MIN	37	48	62	39	14	25
AP_GEOMETRIC	AP_dDFIRE	37	33	44	18	11	7
AP_GEOMETRIC	CP_SKOa	37	52	69	49	17	32
AP_GEOMETRIC	CHARMM_TOT	37	49	62	38	13	25
AP_GEOMETRIC	ROSETTADOCK	37	41	50	22	9	13
AP_GEOMETRIC	ATTRACT	37	38	48	21	10	11
AP_GEOMETRIC	PROPNSTS	37	51	70	52	19	33
AP_GEOMETRIC	DESOLV	37	46	63	43	17	26
AP_GEOMETRIC	CP_Qm	37	49	66	46	17	29
CP_SJKG	AP_T1	51	54	75	45	21	24
CP_SJKG	ZRANK2	51	68	78	37	10	27
CP_SJKG	PISA	51	55	72	38	17	21
CP_SJKG	AP_T2	51	56	74	41	18	23
CP_SJKG	PYDOCK_TOT	51	54	71	37	17	20
CP_SJKG	FIREDOCK_AB	51	47	72	46	25	21
CP_SJKG	FIREDOCK	51	50	73	45	23	22
CP_SJKG	CP_SKOIP	51	56	71	35	15	20
CP_SJKG	CP_BFKV	51	54	65	25	11	14
CP_SJKG	FIREDOCK_EI	51	47	72	46	25	21
CP_SJKG	CP_DECK	51	55	71	36	16	20
CP_SJKG	AP_OPUS_PSP	51	60	74	37	14	23
CP_SJKG	ZRANK	51	43	73	52	30	22
CP_SJKG	CP_BT	51	46	65	33	19	14
CP_SJKG	CP_TSC	51	58	78	47	20	27
CP_SJKG	CP_RMFCA	51	40	68	45	28	17
CP_SJKG	CP_TEl	51	50	70	39	20	19
CP_SJKG	CP_TD	51	49	66	32	17	15
CP_SJKG	CP_MJ3h	51	60	70	29	10	19
CP_SJKG	AP_DARS	51	49	66	32	17	15
CP_SJKG	CP_SKOb	51	51	61	20	10	10
CP_SJKG	CP_TB	51	50	68	35	18	17
CP_SJKG	SPIDER	51	41	72	52	31	21
CP_SJKG	SIPPER	51	56	70	33	14	19
CP_SJKG	AP_DCOMPLEX	51	37	71	54	34	20
CP_SJKG	CP_Qp	51	49	67	34	18	16
CP_SJKG	CP_HLPL	51	52	69	35	17	18
CP_SJKG	CHARMM_ELE	51	39	71	52	32	20
CP_SJKG	CP_MIXRANK_MIN	51	42	63	33	21	12
CP_SJKG	CP_ES3DC_MIN	51	50	66	31	16	15
CP_SJKG	CP_ZS3DC_MIN	51	48	65	31	17	14

Table continued on next page

Table continued from previous page

Method A	Method B	$\|A\|$	$\|B\|$	$\|A \cup B\|$	$\|A \ominus B\|$	$\|A \backslash B\|$	$\|B \backslash A\|$
CP_SJKG	AP_dDFIRE	51	33	67	50	34	16
CP_SJKG	CP_SKOa	51	52	58	13	6	7
CP_SJKG	CHARMM_TOT	51	49	70	40	21	19
CP_SJKG	ROSETTADOCK	51	41	72	52	31	21
CP_SJKG	ATTRACT	51	38	69	49	31	18
CP_SJKG	PROPNSTS	51	51	64	26	13	13
CP_SJKG	DESOLV	51	46	62	27	16	11
CP_SJKG	CP_Qm	51	49	58	16	9	7
CP_SJKG	AP_GEOMETRIC	51	37	69	50	32	18
CP_Qa	AP_T1	44	54	71	44	17	27
CP_Qa	ZRANK2	44	68	77	42	9	33
CP_Qa	PISA	44	55	71	43	16	27
CP_Qa	AP_T2	44	56	74	48	18	30
CP_Qa	PYDOCK_TOT	44	54	69	40	15	25
CP_Qa	FIREDOCK_AB	44	47	73	55	26	29
CP_Qa	FIREDOCK	44	50	73	52	23	29
CP_Qa	CP_SKOIP	44	56	69	38	13	25
CP_Qa	CP_BFKV	44	54	66	34	12	22
CP_Qa	FIREDOCK_EI	44	47	68	45	21	24
CP_Qa	CP_DECK	44	55	67	35	12	23
CP_Qa	AP_OPUS_PSP	44	60	74	44	14	30
CP_Qa	ZRANK	44	43	69	51	26	25
CP_Qa	CP_BT	44	46	65	40	19	21
CP_Qa	CP_TSC	44	58	76	50	18	32
CP_Qa	CP_RMFCA	44	40	65	46	25	21
CP_Qa	CP_TEl	44	50	65	36	15	21
CP_Qa	CP_TD	44	49	65	37	16	21
CP_Qa	CP_MJ3h	44	60	68	32	8	24
CP_Qa	AP_DARS	44	49	65	37	16	21
CP_Qa	CP_SKOb	44	51	65	35	14	21
CP_Qa	CP_TB	44	50	65	36	15	21
CP_Qa	SPIDER	44	41	70	55	29	26
CP_Qa	SIPPER	44	56	71	42	15	27
CP_Qa	AP_DCOMPLEX	44	37	67	53	30	23
CP_Qa	CP_Qp	44	49	66	39	17	22
CP_Qa	CP_HLPL	44	52	68	40	16	24
CP_Qa	CHARMM_ELE	44	39	67	51	28	23
CP_Qa	CP_MIXRANK_MIN	44	42	62	38	20	18
CP_Qa	CP_ES3DC_MIN	44	50	66	38	16	22
CP_Qa	CP_ZS3DC_MIN	44	48	64	36	16	20
CP_Qa	AP_dDFIRE	44	33	65	53	32	21
CP_Qa	CP_SKOa	44	52	62	28	10	18
CP_Qa	CHARMM_TOT	44	49	66	39	17	22
CP_Qa	ROSETTADOCK	44	41	68	51	27	24
CP_Qa	ATTRACT	44	38	67	52	29	23
CP_Qa	PROPNSTS	44	51	62	29	11	18
CP_Qa	DESOLV	44	46	60	30	14	16
CP_Qa	CP_Qm	44	49	53	13	4	9
CP_Qa	AP_GEOMETRIC	44	37	65	49	28	21
CP_Qa	CP_SJKG	44	51	59	23	8	15
CG_ENV	AP_T1	42	54	69	42	15	27
CG_ENV	ZRANK2	42	68	80	50	12	38
CG_ENV	PISA	42	55	73	49	18	31
CG_ENV	AP_T2	42	56	70	42	14	28
CG_ENV	PYDOCK_TOT	42	54	68	40	14	26
CG_ENV	FIREDOCK_AB	42	47	68	47	21	26
CG_ENV	FIREDOCK	42	50	67	42	17	25
CG_ENV	CP_SKOIP	42	56	65	32	9	23
CG_ENV	CP_BFKV	42	54	70	44	16	28
CG_ENV	FIREDOCK_EI	42	47	64	39	17	22
CG_ENV	CP_DECK	42	55	67	37	12	25
CG_ENV	AP_OPUS_PSP	42	60	75	48	15	33
CG_ENV	ZRANK	42	43	67	49	24	25
CG_ENV	CP_BT	42	46	65	42	19	23
CG_ENV	CP_TSC	42	58	73	46	15	31
CG_ENV	CP_RMFCA	42	40	61	40	21	19
CG_ENV	CP_TEl	42	50	65	38	15	23
CG_ENV	CP_TD	42	49	63	35	14	21

Table continued on next page

Table continued from previous page

Method A	Method B	$\lvert A\rvert$	$\lvert B\rvert$	$\lvert A\cup B\rvert$	$\lvert A\ominus B\rvert$	$\lvert A\backslash B\rvert$	$\lvert B\backslash A\rvert$
CG_ENV	CP_MJ3h	42	60	68	34	8	26
CG_ENV	AP_DARS	42	49	59	27	10	17
CG_ENV	CP_SKOb	42	51	68	43	17	26
CG_ENV	CP_TB	42	50	67	42	17	25
CG_ENV	SPIDER	42	41	64	45	23	22
CG_ENV	SIPPER	42	56	73	48	17	31
CG_ENV	AP_DCOMPLEX	42	37	63	47	26	21
CG_ENV	CP_Qp	42	49	69	47	20	27
CG_ENV	CP_HLPL	42	52	70	46	18	28
CG_ENV	CHARMM_ELE	42	39	67	53	28	25
CG_ENV	CP_MIXRANK_MIN	42	42	64	44	22	22
CG_ENV	CP_ES3DC_MIN	42	50	74	56	24	32
CG_ENV	CP_ZS3DC_MIN	42	48	71	52	23	29
CG_ENV	AP_dDFIRE	42	33	58	41	25	16
CG_ENV	CP_SKOa	42	52	70	46	18	28
CG_ENV	CHARMM_TOT	42	49	63	35	14	21
CG_ENV	ROSETTADOCK	42	41	63	43	22	21
CG_ENV	ATTRACT	42	38	65	50	27	23
CG_ENV	PROPNSTS	42	51	69	45	18	27
CG_ENV	DESOLV	42	46	59	30	13	17
CG_ENV	CP_Qm	42	49	66	41	17	24
CG_ENV	AP_GEOMETRIC	42	37	63	47	26	21
CG_ENV	CP_SJKG	42	51	66	39	15	24
CG_ENV	CP_Qa	42	44	59	32	15	17
CP_TEs	AP_T1	41	54	64	33	10	23
CP_TEs	ZRANK2	41	68	73	37	5	32
CP_TEs	PISA	41	55	66	36	11	25
CP_TEs	AP_T2	41	56	68	39	12	27
CP_TEs	PYDOCK_TOT	41	54	66	37	12	25
CP_TEs	FIREDOCK_AB	41	47	66	44	19	25
CP_TEs	FIREDOCK	41	50	66	41	16	25
CP_TEs	CP_SKOIP	41	56	66	35	10	25
CP_TEs	CP_BFKV	41	54	65	35	11	24
CP_TEs	FIREDOCK_EI	41	47	63	38	16	22
CP_TEs	CP_DECK	41	55	63	30	8	22
CP_TEs	AP_OPUS_PSP	41	60	68	35	8	27
CP_TEs	ZRANK	41	43	61	38	18	20
CP_TEs	CP_BT	41	46	62	37	16	21
CP_TEs	CP_TSC	41	58	70	41	12	29
CP_TEs	CP_RMFCA	41	40	58	35	18	17
CP_TEs	CP_TEl	41	50	59	27	9	18
CP_TEs	CP_TD	41	49	59	28	10	18
CP_TEs	CP_MJ3h	41	60	65	29	5	24
CP_TEs	AP_DARS	41	49	67	44	18	26
CP_TEs	CP_SKOb	41	51	63	34	12	22
CP_TEs	CP_TB	41	50	64	37	14	23
CP_TEs	SPIDER	41	41	64	46	23	23
CP_TEs	SIPPER	41	56	67	37	11	26
CP_TEs	AP_DCOMPLEX	41	37	60	42	23	19
CP_TEs	CP_Qp	41	49	62	34	13	21
CP_TEs	CP_HLPL	41	52	63	33	11	22
CP_TEs	CHARMM_ELE	41	39	60	40	21	19
CP_TEs	CP_MIXRANK_MIN	41	42	56	29	14	15
CP_TEs	CP_ES3DC_MIN	41	50	63	35	13	22
CP_TEs	CP_ZS3DC_MIN	41	48	61	33	13	20
CP_TEs	AP_dDFIRE	41	33	56	38	23	15
CP_TEs	CP_SKOa	41	52	65	37	13	24
CP_TEs	CHARMM_TOT	41	49	68	46	19	27
CP_TEs	ROSETTADOCK	41	41	62	42	21	21
CP_TEs	ATTRACT	41	38	57	35	19	16
CP_TEs	PROPNSTS	41	51	63	34	12	22
CP_TEs	DESOLV	41	46	63	39	17	22
CP_TEs	CP_Qm	41	49	63	36	14	22
CP_TEs	AP_GEOMETRIC	41	37	58	38	21	17
CP_TEs	CP_SJKG	41	51	64	36	13	23
CP_TEs	CP_Qa	41	44	58	31	14	17
CP_TEs	CG_ENV	41	42	60	37	18	19
AP_GOAP_ALL	AP_T1	40	54	69	44	15	29

Table continued on next page

Table continued from previous page

Method A	Method B	$\vert A\vert$	$\vert B\vert$	$\vert A\cup B\vert$	$\vert A\ominus B\vert$	$\vert A\backslash B\vert$	$\vert B\backslash A\vert$
AP_GOAP_ALL	ZRANK2	40	68	76	44	8	36
AP_GOAP_ALL	PISA	40	55	66	37	11	26
AP_GOAP_ALL	AP_T2	40	56	66	36	10	26
AP_GOAP_ALL	PYDOCK_TOT	40	54	68	42	14	28
AP_GOAP_ALL	FIREDOCK_AB	40	47	66	45	19	26
AP_GOAP_ALL	FIREDOCK	40	50	66	42	16	26
AP_GOAP_ALL	CP_SKOIP	40	56	67	38	11	27
AP_GOAP_ALL	CP_BFKV	40	54	70	46	16	30
AP_GOAP_ALL	FIREDOCK_EI	40	47	63	39	16	23
AP_GOAP_ALL	CP_DECK	40	55	67	39	12	27
AP_GOAP_ALL	AP_OPUS_PSP	40	60	69	38	9	29
AP_GOAP_ALL	ZRANK	40	43	65	47	22	25
AP_GOAP_ALL	CP_BT	40	46	62	38	16	22
AP_GOAP_ALL	CP_TSC	40	58	71	44	13	31
AP_GOAP_ALL	CP_RMFCA	40	40	59	38	19	19
AP_GOAP_ALL	CP_TEl	40	50	64	38	14	24
AP_GOAP_ALL	CP_TD	40	49	62	35	13	22
AP_GOAP_ALL	CP_MJ3h	40	60	68	36	8	28
AP_GOAP_ALL	AP_DARS	40	49	58	27	9	18
AP_GOAP_ALL	CP_SKOb	40	51	66	41	15	26
AP_GOAP_ALL	CP_TB	40	50	67	44	17	27
AP_GOAP_ALL	SPIDER	40	41	60	39	19	20
AP_GOAP_ALL	SIPPER	40	56	69	42	13	29
AP_GOAP_ALL	AP_DCOMPLEX	40	37	61	45	24	21
AP_GOAP_ALL	CP_Qp	40	49	67	45	18	27
AP_GOAP_ALL	CP_HLPL	40	52	67	42	15	27
AP_GOAP_ALL	CHARMM_ELE	40	39	66	53	27	26
AP_GOAP_ALL	CP_MIXRANK_MIN	40	42	65	48	23	25
AP_GOAP_ALL	CP_ES3DC_MIN	40	50	71	52	21	31
AP_GOAP_ALL	CP_ZS3DC_MIN	40	48	70	52	22	30
AP_GOAP_ALL	AP_dDFIRE	40	33	56	39	23	16
AP_GOAP_ALL	CP_SKOa	40	52	65	38	13	25
AP_GOAP_ALL	CHARMM_TOT	40	49	65	41	16	25
AP_GOAP_ALL	ROSETTADOCK	40	41	61	41	20	21
AP_GOAP_ALL	ATTRACT	40	38	62	46	24	22
AP_GOAP_ALL	PROPNSTS	40	51	68	45	17	28
AP_GOAP_ALL	DESOLV	40	46	57	28	11	17
AP_GOAP_ALL	CP_Qm	40	49	65	41	16	25
AP_GOAP_ALL	AP_GEOMETRIC	40	37	61	45	24	21
AP_GOAP_ALL	CP_SJKG	40	51	63	35	12	23
AP_GOAP_ALL	CP_Qa	40	44	61	38	17	21
AP_GOAP_ALL	CG_ENV	40	42	55	28	13	15
AP_GOAP_ALL	CP_TEs	40	41	61	41	20	21
AP_DDGau	AP_T1	31	54	64	43	10	33
AP_DDGau	ZRANK2	31	68	71	43	3	40
AP_DDGau	PISA	31	55	61	36	6	30
AP_DDGau	AP_T2	31	56	65	43	9	34
AP_DDGau	PYDOCK_TOT	31	54	66	47	12	35
AP_DDGau	FIREDOCK_AB	31	47	56	34	9	25
AP_DDGau	FIREDOCK	31	50	61	41	11	30
AP_DDGau	CP_SKOIP	31	56	71	55	15	40
AP_DDGau	CP_BFKV	31	54	67	49	13	36
AP_DDGau	FIREDOCK_EI	31	47	60	42	13	29
AP_DDGau	CP_DECK	31	55	67	48	12	36
AP_DDGau	AP_OPUS_PSP	31	60	66	41	6	35
AP_DDGau	ZRANK	31	43	53	32	10	22
AP_DDGau	CP_BT	31	46	60	43	14	29
AP_DDGau	CP_TSC	31	58	65	41	7	34
AP_DDGau	CP_RMFCA	31	40	52	33	12	21
AP_DDGau	CP_TEl	31	50	60	39	10	29
AP_DDGau	CP_TD	31	49	60	40	11	29
AP_DDGau	CP_MJ3h	31	60	69	47	9	38
AP_DDGau	AP_DARS	31	49	67	54	18	36
AP_DDGau	CP_SKOb	31	51	63	44	12	32
AP_DDGau	CP_TB	31	50	60	39	10	29
AP_DDGau	SPIDER	31	41	55	38	14	24
AP_DDGau	SIPPER	31	56	69	51	13	38
AP_DDGau	AP_DCOMPLEX	31	37	48	28	11	17

Table continued on next page

Table continued from previous page

Method A	Method B	$\lvert A\rvert$	$\lvert B\rvert$	$\lvert A\cup B\rvert$	$\lvert A\ominus B\rvert$	$\lvert A\backslash B\rvert$	$\lvert B\backslash A\rvert$
AP_DDGau	CP_Qp	31	49	56	32	7	25
AP_DDGau	CP_HLPL	31	52	60	37	8	29
AP_DDGau	CHARMM_ELE	31	39	55	40	16	24
AP_DDGau	CP_MIXRANK_MIN	31	42	61	49	19	30
AP_DDGau	CP_ES3DC_MIN	31	50	67	53	17	36
AP_DDGau	CP_ZS3DC_MIN	31	48	65	51	17	34
AP_DDGau	AP_dDFIRE	31	33	47	30	14	16
AP_DDGau	CP_SKOa	31	52	66	49	14	35
AP_DDGau	CHARMM_TOT	31	49	63	46	14	32
AP_DDGau	ROSETTADOCK	31	41	54	36	13	23
AP_DDGau	ATTRACT	31	38	49	29	11	18
AP_DDGau	PROPNSTS	31	51	66	50	15	35
AP_DDGau	DESOLV	31	46	64	51	18	33
AP_DDGau	CP_Qm	31	49	64	48	15	33
AP_DDGau	AP_GEOMETRIC	31	37	49	30	12	18
AP_DDGau	CP_SJKG	31	51	67	52	16	36
AP_DDGau	CP_Qa	31	44	63	51	19	32
AP_DDGau	CG_ENV	31	42	63	53	21	32
AP_DDGau	CP_TEs	31	41	56	40	15	25
AP_DDGau	AP_GOAP_ALL	31	40	60	49	20	29
ODA	AP_T1	33	54	63	39	9	30
ODA	ZRANK2	33	68	74	47	6	41
ODA	PISA	33	55	65	42	10	32
ODA	AP_T2	33	56	65	41	9	32
ODA	PYDOCK_TOT	33	54	63	39	9	30
ODA	FIREDOCK_AB	33	47	63	46	16	30
ODA	FIREDOCK	33	50	64	45	14	31
ODA	CP_SKOIP	33	56	66	43	10	33
ODA	CP_BFKV	33	54	70	53	16	37
ODA	FIREDOCK_EI	33	47	61	42	14	28
ODA	CP_DECK	33	55	65	42	10	32
ODA	AP_OPUS_PSP	33	60	65	37	5	32
ODA	ZRANK	33	43	58	40	15	25
ODA	CP_BT	33	46	63	47	17	30
ODA	CP_TSC	33	58	66	41	8	33
ODA	CP_RMFCA	33	40	54	35	14	21
ODA	CP_TEl	33	50	67	51	17	34
ODA	CP_TD	33	49	61	40	12	28
ODA	CP_MJ3h	33	60	70	47	10	37
ODA	AP_DARS	33	49	63	44	14	30
ODA	CP_SKOb	33	51	67	50	16	34
ODA	CP_TB	33	50	64	45	14	31
ODA	SPIDER	33	41	53	32	12	20
ODA	SIPPER	33	56	62	35	6	29
ODA	AP_DCOMPLEX	33	37	51	32	14	18
ODA	CP_Qp	33	49	60	38	11	27
ODA	CP_HLPL	33	52	62	39	10	29
ODA	CHARMM_ELE	33	39	62	52	23	29
ODA	CP_MIXRANK_MIN	33	42	58	41	16	25
ODA	CP_ES3DC_MIN	33	50	67	51	17	34
ODA	CP_ZS3DC_MIN	33	48	65	49	17	32
ODA	AP_dDFIRE	33	33	45	24	12	12
ODA	CP_SKOa	33	52	69	53	17	36
ODA	CHARMM_TOT	33	49	65	48	16	32
ODA	ROSETTADOCK	33	41	56	38	15	23
ODA	ATTRACT	33	38	51	31	13	18
ODA	PROPNSTS	33	51	70	56	19	37
ODA	DESOLV	33	46	57	35	11	24
ODA	CP_Qm	33	49	66	50	17	33
ODA	AP_GEOMETRIC	33	37	50	30	13	17
ODA	CP_SJKG	33	51	66	48	15	33
ODA	CP_Qa	33	44	63	49	19	30
ODA	CG_ENV	33	42	58	41	16	25
ODA	CP_TEs	33	41	56	38	15	23
ODA	AP_GOAP_ALL	33	40	54	35	14	21
ODA	AP_DDGau	33	31	53	42	22	20
ELE	AP_T1	39	54	64	35	10	25
ELE	ZRANK2	39	68	74	41	6	35

Table continued on next page

Table continued from previous page

Method A	Method B	$\|A\|$	$\|B\|$	$\|A \cup B\|$	$\|A \ominus B\|$	$\|A \backslash B\|$	$\|B \backslash A\|$
ELE	PISA	39	55	66	38	11	27
ELE	AP_T2	39	56	67	39	11	28
ELE	PYDOCK_TOT	39	54	62	31	8	23
ELE	FIREDOCK_AB	39	47	58	30	11	19
ELE	FIREDOCK	39	50	61	33	11	22
ELE	CP_SKOIP	39	56	74	53	18	35
ELE	CP_BFKV	39	54	68	43	14	29
ELE	FIREDOCK_EI	39	47	62	38	15	23
ELE	CP_DECK	39	55	70	46	15	31
ELE	AP_OPUS_PSP	39	60	72	45	12	33
ELE	ZRANK	39	43	55	28	12	16
ELE	CP_BT	39	46	62	39	16	23
ELE	CP_TSC	39	58	70	43	12	31
ELE	CP_RMFCA	39	40	55	31	15	16
ELE	CP_TEl	39	50	64	39	14	25
ELE	CP_TD	39	49	63	38	14	24
ELE	CP_MJ3h	39	60	71	43	11	32
ELE	AP_DARS	39	49	68	48	19	29
ELE	CP_SKOb	39	51	66	42	15	27
ELE	CP_TB	39	50	61	33	11	22
ELE	SPIDER	39	41	58	36	17	19
ELE	SIPPER	39	56	70	45	14	31
ELE	AP_DCOMPLEX	39	37	54	32	17	15
ELE	CP_Qp	39	49	63	38	14	24
ELE	CP_HLPL	39	52	64	37	12	25
ELE	CHARMM_ELE	39	39	51	24	12	12
ELE	CP_MIXRANK_MIN	39	42	62	43	20	23
ELE	CP_ES3DC_MIN	39	50	67	45	17	28
ELE	CP_ZS3DC_MIN	39	48	64	41	16	25
ELE	AP_dDFIRE	39	33	51	30	18	12
ELE	CP_SKOa	39	52	69	47	17	30
ELE	CHARMM_TOT	39	49	66	44	17	27
ELE	ROSETTADOCK	39	41	58	36	17	19
ELE	ATTRACT	39	38	54	31	16	15
ELE	PROPNSTS	39	51	69	48	18	30
ELE	DESOLV	39	46	64	43	18	25
ELE	CP_Qm	39	49	68	48	19	29
ELE	AP_GEOMETRIC	39	37	57	38	20	18
ELE	CP_SJKG	39	51	69	48	18	30
ELE	CP_Qa	39	44	66	49	22	27
ELE	CG_ENV	39	42	64	47	22	25
ELE	CP_TEs	39	41	59	38	18	20
ELE	AP_GOAP_ALL	39	40	63	47	23	24
ELE	AP_DDGau	39	31	51	32	20	12
ELE	ODA	39	33	58	44	25	19
AP_MPS	AP_T1	40	54	72	50	18	32
AP_MPS	ZRANK2	40	68	74	40	6	34
AP_MPS	PISA	40	55	68	41	13	28
AP_MPS	AP_T2	40	56	72	48	16	32
AP_MPS	PYDOCK_TOT	40	54	73	52	19	33
AP_MPS	FIREDOCK_AB	40	47	67	47	20	27
AP_MPS	FIREDOCK	40	50	70	50	20	30
AP_MPS	CP_SKOIP	40	56	69	42	13	29
AP_MPS	CP_BFKV	40	54	67	40	13	27
AP_MPS	FIREDOCK_EI	40	47	71	55	24	31
AP_MPS	CP_DECK	40	55	70	45	15	30
AP_MPS	AP_OPUS_PSP	40	60	70	40	10	30
AP_MPS	ZRANK	40	43	64	45	21	24
AP_MPS	CP_BT	40	46	64	42	18	24
AP_MPS	CP_TSC	40	58	71	44	13	31
AP_MPS	CP_RMFCA	40	40	60	40	20	20
AP_MPS	CP_TEl	40	50	68	46	18	28
AP_MPS	CP_TD	40	49	65	41	16	25
AP_MPS	CP_MJ3h	40	60	75	50	15	35
AP_MPS	AP_DARS	40	49	68	47	19	28
AP_MPS	CP_SKOb	40	51	60	29	9	20
AP_MPS	CP_TB	40	50	65	40	15	25
AP_MPS	SPIDER	40	41	67	53	26	27

Table continued on next page

Table continued from previous page

Method A	Method B	$\|A\|$	$\|B\|$	$\|A \cup B\|$	$\|A \ominus B\|$	$\|A \backslash B\|$	$\|B \backslash A\|$
AP_MPS	SIPPER	40	56	68	40	12	28
AP_MPS	AP_DCOMPLEX	40	37	60	43	23	20
AP_MPS	CP_Qp	40	49	58	27	9	18
AP_MPS	CP_HLPL	40	52	61	30	9	21
AP_MPS	CHARMM_ELE	40	39	60	41	21	20
AP_MPS	CP_MIXRANK_MIN	40	42	58	34	16	18
AP_MPS	CP_ES3DC_MIN	40	50	62	34	12	22
AP_MPS	CP_ZS3DC_MIN	40	48	61	34	13	21
AP_MPS	AP_dDFIRE	40	33	57	41	24	17
AP_MPS	CP_SKOa	40	52	62	32	10	22
AP_MPS	CHARMM_TOT	40	49	72	55	23	32
AP_MPS	ROSETTADOCK	40	41	64	47	23	24
AP_MPS	ATTRACT	40	38	61	44	23	21
AP_MPS	PROPNSTS	40	51	60	29	9	20
AP_MPS	DESOLV	40	46	65	44	19	25
AP_MPS	CP_Qm	40	49	62	35	13	22
AP_MPS	AP_GEOMETRIC	40	37	60	43	23	20
AP_MPS	CP_SJKG	40	51	63	35	12	23
AP_MPS	CP_Qa	40	44	61	38	17	21
AP_MPS	CG_ENV	40	42	68	54	26	28
AP_MPS	CP_TEs	40	41	60	39	19	20
AP_MPS	AP_GOAP_ALL	40	40	67	54	27	27
AP_MPS	AP_DDGau	40	31	54	37	23	14
AP_MPS	ODA	40	33	61	49	28	21
AP_MPS	ELE	40	39	61	43	22	21
AA_PROP	AP_T1	32	54	68	50	14	36
AA_PROP	ZRANK2	32	68	79	58	11	47
AA_PROP	PISA	32	55	67	47	12	35
AA_PROP	AP_T2	32	56	69	50	13	37
AA_PROP	PYDOCK_TOT	32	54	67	48	13	35
AA_PROP	FIREDOCK_AB	32	47	62	45	15	30
AA_PROP	FIREDOCK	32	50	63	44	13	31
AA_PROP	CP_SKOIP	32	56	68	48	12	36
AA_PROP	CP_BFKV	32	54	70	54	16	38
AA_PROP	FIREDOCK_EI	32	47	60	41	13	28
AA_PROP	CP_DECK	32	55	68	49	13	36
AA_PROP	AP_OPUS_PSP	32	60	72	52	12	40
AA_PROP	ZRANK	32	43	58	41	15	26
AA_PROP	CP_BT	32	46	64	50	18	32
AA_PROP	CP_TSC	32	58	71	52	13	39
AA_PROP	CP_RMFCA	32	40	57	42	17	25
AA_PROP	CP_TEl	32	50	63	44	13	31
AA_PROP	CP_TD	32	49	64	47	15	32
AA_PROP	CP_MJ3h	32	60	68	44	8	36
AA_PROP	AP_DARS	32	49	61	41	12	29
AA_PROP	CP_SKOb	32	51	69	55	18	37
AA_PROP	CP_TB	32	50	65	48	15	33
AA_PROP	SPIDER	32	41	56	39	15	24
AA_PROP	SIPPER	32	56	69	50	13	37
AA_PROP	AP_DCOMPLEX	32	37	55	41	18	23
AA_PROP	CP_Qp	32	49	63	45	14	31
AA_PROP	CP_HLPL	32	52	65	46	13	33
AA_PROP	CHARMM_ELE	32	39	63	55	24	31
AA_PROP	CP_MIXRANK_MIN	32	42	60	46	18	28
AA_PROP	CP_ES3DC_MIN	32	50	65	48	15	33
AA_PROP	CP_ZS3DC_MIN	32	48	65	50	17	33
AA_PROP	AP_dDFIRE	32	33	52	39	19	20
AA_PROP	CP_SKOa	32	52	69	54	17	37
AA_PROP	CHARMM_TOT	32	49	63	45	14	31
AA_PROP	ROSETTADOCK	32	41	57	41	16	25
AA_PROP	ATTRACT	32	38	55	40	17	23
AA_PROP	PROPNSTS	32	51	70	57	19	38
AA_PROP	DESOLV	32	46	60	42	14	28
AA_PROP	CP_Qm	32	49	64	47	15	32
AA_PROP	AP_GEOMETRIC	32	37	56	43	19	24
AA_PROP	CP_SJKG	32	51	67	51	16	35
AA_PROP	CP_Qa	32	44	60	44	16	28
AA_PROP	CG_ENV	32	42	59	44	17	27

Table continued on next page

Table continued from previous page

Method A	Method B	$\|A\|$	$\|B\|$	$\|A \cup B\|$	$\|A \ominus B\|$	$\|A \backslash B\|$	$\|B \backslash A\|$
AA_PROP	CP_TEs	32	41	58	43	17	26
AA_PROP	AP_GOAP_ALL	32	40	57	42	17	25
AA_PROP	AP_DDGau	32	31	52	41	21	20
AA_PROP	ODA	32	33	54	43	21	22
AA_PROP	ELE	32	39	59	47	20	27
AA_PROP	AP_MPS	32	40	61	50	21	29
